# Controlled Lactonization of *o*-Coumaric Esters Mediated by Supramolecular Gels

**DOI:** 10.3390/gels9040350

**Published:** 2023-04-21

**Authors:** Fabia Cenciarelli, Giuseppe Falini, Demetra Giuri, Claudia Tomasini

**Affiliations:** Dipartimento di Chimica Giacomo Ciamician, Università di Bologna, Via Selmi, 2, 40126 Bologna, Italy

**Keywords:** lactonization, L-DOPA, low molecular weight gelators, profragrances, solar light, supramolecular gels, xerogels

## Abstract

Fragrances are volatile organic compounds widely used in our daily life. Unfortunately, the high volatility required to reach human receptors reduces their persistency in the air. To contrast this effect, several strategies may be used. Among them, we present here the combination of two techniques: the microencapsulation in supramolecular gels and the use of profragrances. We report a study on the controlled lactonization of four esters derived from *o*-coumaric acid. The ester lactonization spontaneously occurs after exposure to solar light, releasing coumarin and the corresponding alcohol. To determine the rate of fragrance release, we compared the reaction in solution and in a supramolecular gel and we demonstrated that the lactonization reaction always occurs slower in the gel. We also studied the more suitable gel for this aim, by comparing the properties of two supramolecular gels obtained with the gelator Boc-L-DOPA(Bn)_2_-OH in a 1:1 ethanol/water mixture in different gelator concentration (0.2% and 1% *w*/*v*). The gel prepared with 1% *w/v* gelator concentration is stronger and less transparent than the other and was used for the profragrances encapsulation. In any case, we obtained a significative reduction of lactonization reaction in gel, compared with the same reaction in solution.

## 1. Introduction

Fragrances are volatile organic compounds widely used in our daily life, as smell is a powerful trigger for the subconscious and awakens emotions [1,2]. Biologically, volatile molecules are recognized by all species and used as a means of communication, so it is important to develop their application to facilitate human interactions [3]. Unfortunately, the high volatility required to reach the human receptors means that these molecules are not persistent in the air [4,5] The need to increase the persistency of olfactive perception prompted the researchers to design several strategies. Among them, microencapsulation is widely used in the cosmetic, food, agriculture, and pharmaceutical industries, [6,7] as the encapsulation of a specific substance can not only enhance its stability against degradation, but can also allow a controlled release of the substance in a specific medium [2,8]. The use of gels fulfils all these requirements and may be certainly applied for microencapsulation. In particular, supramolecular gels based on low-molecular-weight gelators (LMWGs) have recently attracted great attention due to their wide applicability in many fields, being used in optoelectronics [9], nanomaterials, shape memories, templated synthesis and crystallization, [10] as well as drug carriers and cell culture medium [11,12,13,14]. The formation of LMW gels is driven by weak noncovalent interactions, such as ion−ion, H-bonding, π−π stacking, and van der Waals [15,16]. These interactions facilitate the generation of a 3D network of self-assembled fibers that immobilize the solvent within the entangled supramolecular structure, leading to the gel formation. LMW peptide gelators offer many advantages compared to polymers. Their chemical structure is easily tuneable, they can respond to different stimuli (triggers) to form/disrupt the gel network, and they are often biocompatible and biodegradable [17,18,19,20,21]. The controlled release of fragrances from gel media has been reported by us [22] and other research groups [6,8,23,24,25,26,27,28,29,30,31,32].

The use of profragrances is another strategy to increase the persistency of volatile fragrances. Profragrances are non-volatile and odorless molecules which release the volatile fragrances by bond cleavage [33,34,35,36,37]. Specific reaction conditions, such as hydrolysis, temperature changes, as well as the action of light, oxygen, enzymes, or microorganisms, can be used to liberate the many different chemical functionalities. For example, Schiff bases have been studied because they have a low volatility and, at the same time, they can be hydrolyzed in an acidic environment [22,38,39,40]. The hydrolysis is a crucial step and by regulating it, it is possible to prolong or shorten the time in which the fragrance is perceived. Oxygen was also considered as a possible trigger for profragrance modification, because of its reactivity with labile groups (for example aldehydes) which also alters products during prolonged storage. However, because of oxygen ubiquity, oxygen-sensitive precursors can undergo continuous and uncontrolled oxidation reactions rather than a triggered release in a specific application. This is the reason why a very limited number of oxygen-sensitive fragrance delivery systems have been reported so far [41,42].

Another example is the UVA-sensitive photocleavable delivery systems. Since the surfaces on which the evaporation of the volatiles takes place are usually exposed to natural daylight, photoresponsive delivery systems were found to be particularly appropriate to control the release of bioactive volatile compounds.

As the aim of both profragrances and encapsulating medium is to release fragrances in a given time to extend their persistency in the air, the combination of these two techniques may significantly increase the release time and the persistency of the fragrance. Starting from this assumption, we studied the controlled cleavage of a group of profragrances that release coumarin and selected alcohols by lactonization catalyzed by solar light. In this project, four photolabile profragrances were selected as they easily undergo light-driven lactonization. All of them were successfully synthesized, purified, and characterized. Besides the kinetics of fragrance release from the profragrances, we investigated the role of the encapsulation of these compounds in supramolecular gels made from peptide based LMWGs.

## 2. Results and Discussion

**Figure 1:**
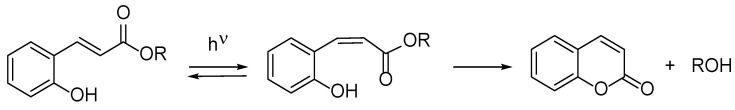
Photoisomerization and lactonization of *E*-*o*-hydroxy cinnamates. Substituents on the aromatic rings are omitted for the sake of clarity.

The general reaction for profragrance lactonization is reported in Figure 1. This process was successfully studied in solution on Scentaurus Tonkarose, [43] a profragrance triggered by light. Exposure to solar light, and in particular to the UVA portion of the spectrum, provides the necessary energy to induce (*E*)/(*Z*)-isomerization followed by a spontaneous lactonization reaction, which leads to the release of coumarin and the perfumed alcohol. This reaction was completed in solution in 15 min. The potential of (*E*)-*o*-hydroxycinnamates has motivated researchers to put more effort on searching for more profragrances as well as establishing methodologies to incorporate them in different products in the past few decades.

The study of the controlled photo-cleavage of this class of profragrances requires the preliminary analysis of two aspects: the synthesis and characterization of the reagents and the preparation of the gel that entraps the molecules. Then, the behavior of entrapped profragrances exposed to solar light is studied.

### 2.1. Profragrances Preparation

**Scheme 1:**
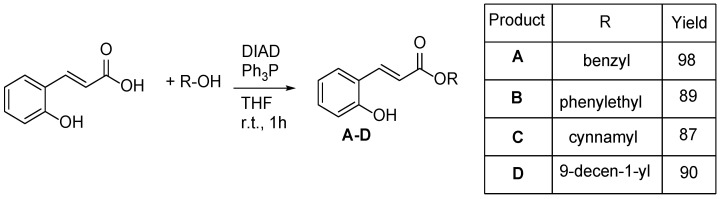
Schematic representation of the preparation of the profragrances **A**–**D**.

Profragrances **A**–**D** were prepared by Mitsunobu esterification [44] from *o*-hydroxy cynnamic acid and four alcohols as depicted in Scheme 1. The reactions proceed in 1 h, with the formation of the products as white solids in high yields, ranging between 87% and 98% after purification obtained by silica gel chromatography. We chose these alcohols as they are all fragrances widely used in perfumery and show different chemical structures so that we can also study the effect of the alcohol structure on the kinetic of the lactonization.

### 2.2. Gel Characterization

Then we studied the conditions for the formation of gels that may be employed to trap the molecules. The ideal gel should increase the time of coumarin formation from compounds **A**–**D**, without interfering with this process with side reactions.

**Figure 2:**
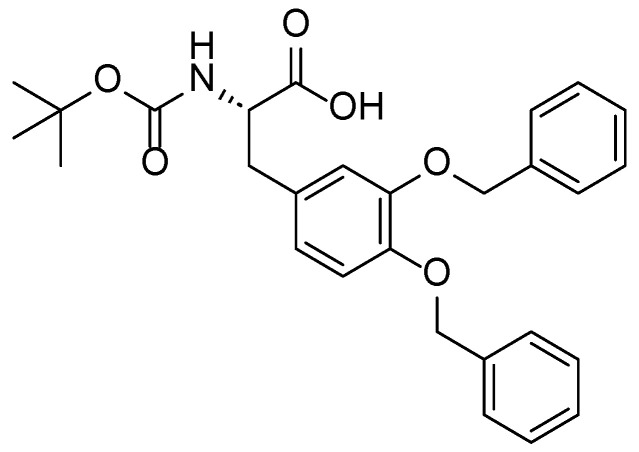
Chemical structure of the gelator Boc-L-DOPA(Bn)_2_-OH.

For this aim, we prepared some gels using Boc-L-DOPA(Bn)_2_-OH [45,46], a gelator widely used in our research lab (Figure 2) [47]. This gelator can form a variety of noncovalent interactions (π-π stackings between the aromatic rings and H-bonds in the carbamate and carboxylic acid portions, as illustrated in Figure S1) and demonstrated its robustness forming gels under several conditions and in the presence of different fillers [10,48,49,50].

In this work, the gels were prepared in a 1:1 mixture of water and ethanol, a suitable solvent for both gel formation and for the solubilization of the synthesized profragrances. A preliminary study was performed to determine the minimum gelation concentration (MGC), which resulted to be 0.2% *w*/*v* (**G02**), as at lower concentrations no gel formed (Figure S2). Then, we prepared two gels, **G02** and **G1** (containing the gelator in 1.0% *w/v* concentration in 1:1 EtOH/H_2_O) and we analyzed their properties with several techniques to compare them and to establish which is the most suitable to study the effect of gelator concentration on the kinetics of lactonization and fragrance release.

**Figure 3:**
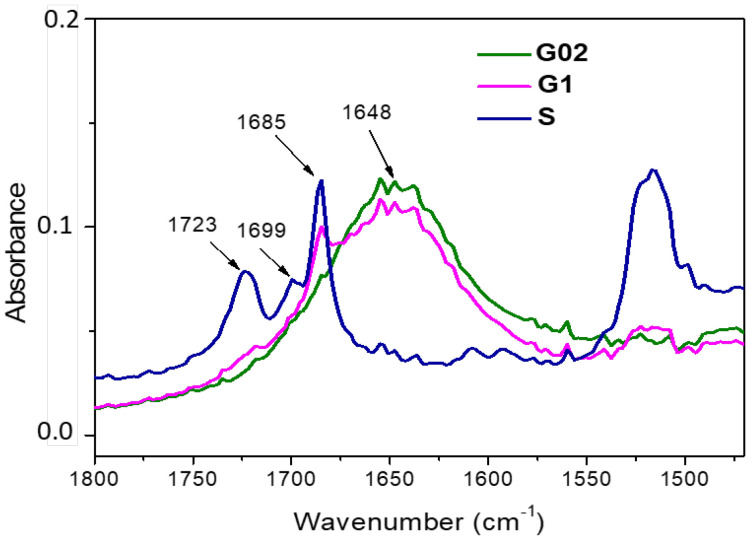
Inset of the FT-IR spectra of the gels **G1** and **G02**, compared with a solution **S** of the gelator Boc-L-DOPA(Bn)_2_-OH.

To study the formation of the fibers which constitute the gel, we analyzed samples **G02** and **G1** by FT-IR spectroscopy (Figure 3 and Figure 3 and Figure S3), together with the gelator solution in ethanol. The analyses of the FT-IR spectra of the solution and gels, as well as the comparison between the peaks that have appeared or disappeared, allows to gather information about the driving forces of the hydrogelation process [51,52,53]. In the region between 1800 and 1500 cm^−1^, the peaks positions of the carbonyl of the gelators **G02** and **G1** have wavenumbers lower than those of the peaks of the solution **S**. In particular, the peaks at 1723 and at 1699 cm^−1^ recorded for **S** are totally absent in both **G02** and **G1**, while the peak at 1685 cm^−1^ of **S** is totally absent in **G02** while slightly visible in **G1**, and in both gels replaced with a large peak centered at 1648 cm^−1^. This effect may be ascribed to the formation of hydrogen bonds in the fibers, which is complete for **G02**, while a residual amount of free gelator (peak at 1685 cm^−1^) is still present in **G1**. This outcome is confirmed by a morphological analysis of the samples, performed with an optical microscope on wet samples, which reveals that they both have a fibrous structure, denser for sample **G1** than for **G02** (Figure S4).

**Figure 4:**
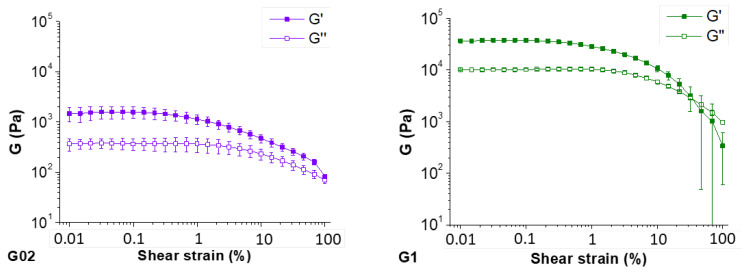
Amplitude sweep analyses of **G02** (**left**) and **G1** (**right**). The experiments were repeated in triplicate and results are expressed as mean ± standard deviation.

The viscoelastic properties of the two samples of gels were measured with a rheometer. The measurement of the amplitude sweep of **G02** and **G1** demonstrated that they are both gels as the G′ modulus is always higher than G″, although **G1** is far stiffer than **G02** (Figure 4 and Table S1). Both samples have a significant elasticity, confirmed by the long linear viscoelastic range (LVER). It is worth noticing that **G02** presents a remarkable elasticity, as the crossover point (breaking point of the gel network) is at 100% of the range of shear strain studied.

**Figure 5:**
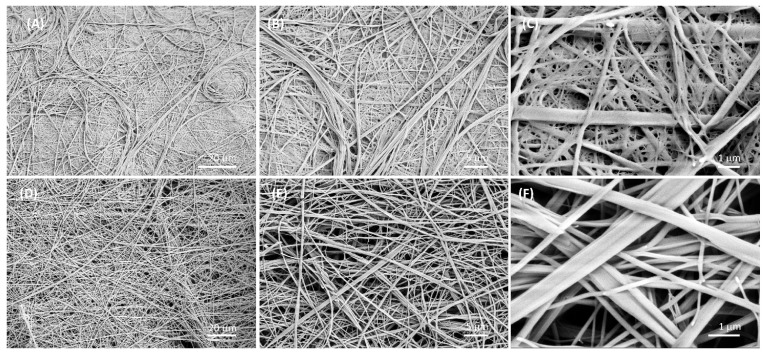
SEM images of the xerogels at increasing magnifications: (**A**–**C**) **XG1** xerogel and (**D**–**F**) **XG02** xerogel.

To get further information on the structure of **G02** and **G1**, we prepared the corresponding xerogels **XG02** and **XG1** and we analyzed them by scanning electron microscopy (SEM) and X-ray powder diffraction (XRD). In Figure 5, the images from **XG1** (top) and **XG02** (bottom) are reported. The low magnification images show that in both samples extremely long fibers were present. They were up to several hundred of micro-meters long with a thickness not higher than 1 µm, thus having a very high aspect ratio. While in **XG02** the fibers are mainly single, in **XG1** many of them are associated in bundles. The high magnification images confirmed this observation and revealed additional differences between the two samples. In **XG02**, only fibrous material is present, and the fibers show a minimal branching and entanglement. Diversely, in **XG1**, the fibers are highly branched and entangled. Moreover, a non-fibrous material, such as thin films, is connecting the fibers. The greater presence of branching and entanglement in **XG1** than in **XG02** agrees with the higher mechanical parameters of **G1** than **G02**, while the interconnecting films are probably generated by the drying process.

**Figure 6:**
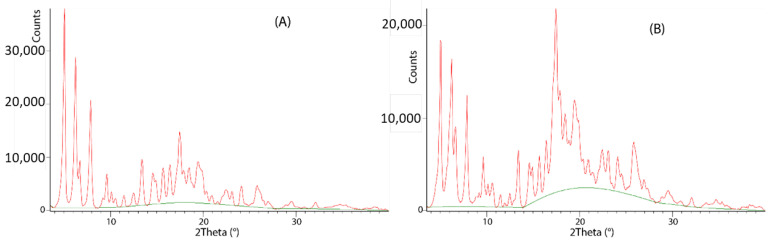
XRD profiles of the xerogel **XG02** (**A**) and the xerogel **XG1**. (**B**) The red and green lines show the diffraction pattern and the amorphous band, respectively.

Additional information on **XG02** and **XG1** xerogels was provided by the analysis of the XRD diffraction patterns (Figure 6). The two patterns show exactly the same diffraction peaks, indicating that the same crystalline material is present in the two xerogels. The diverse diffraction intensities could be due to the different xerogel macrostructure (see SEM observations), which could not be completely removed by the powdering process. The diffraction profiles also show that a higher content of amorphous material is present in **XG1** than **XG02**. This is revealed by the broad band around 2theta of 20°, indicated by the green line in Figure 6. The analysis of the integrated diffraction intensities of the XRD profiles indicates that in the sample **XG1** there is approximately 7 wt.% of amorphous phase. This phase could be associated with unreacted molecules (see FTIR analyses) and form the thin films connecting the fibers in **XG1** (see SEM images).

These results demonstrate that in both cases fibers are formed, but their branching and entanglement vary according to the starting gelator concentration. This effect may strongly affect the time for the light-driven controlled release of fragrances, as a denser network may better entrap the profragrance, interfering with the solar light catalyzed lactonization reaction and prolonging the time of release. For this reason, we compared the kinetics of the lactonization in two gels (**G02-A** and **G1-A**), both containing the profragrance **A** in 2.5 mg/mL in ethanol/water 1:1 mixture and varying the gelator concentration according to the previous results. We also analyzed the kinetics of lactonization for a solution containing only the profragrance **A** in 2.5 mg/mL in ethanol/water 1:1 mixture (**S-A**). The kinetic of the reaction was observed for all the samples after the exposure to a light beam from a solar lamp (for details, see Section 4).

We prepared the three samples **G02**-**A**, **G1**-**A,** and **S-A** both in quartz cuvettes and in glass vials, to also check the impact of the cell material and geometry on the lactonization kinetics. The two gels readily formed under the same conditions reported for **G02** and **G1**, as **A** is completely soluble in the solvent mixture and has no impact on the gelation process.

**Figure 7:**
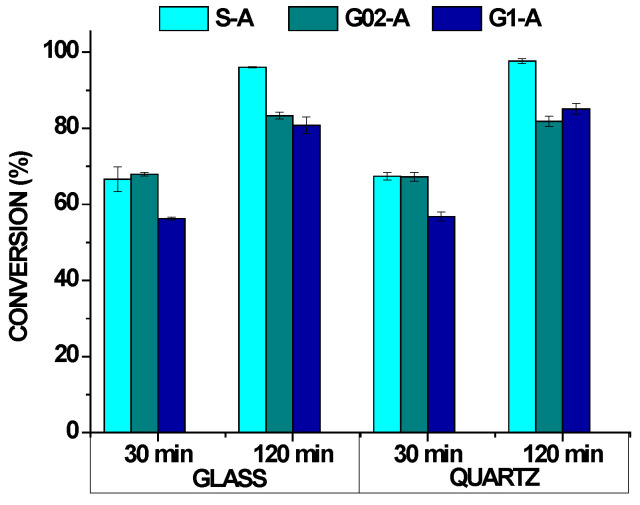
Kinetics of the lactonization of profragrance **A** under selected conditions using the solar lamp. The experiments were repeated in triplicate and results are expressed as mean ± standard deviation.

The release was observed at 30 and 120 min and conversions were calculated on the disappearance of **A**, using a calibration curve (Figures S5 and S6) made by injecting the standard solutions of the profragrances into HPLC-MS (Figure 7 and Table S2). The results demonstrate that using a quartz cuvette or a glass vial has only a modest effect on the kinetic. In contrast, the gelator concentration has a strong impact on the kinetic, mainly at 30 min, showing that the denser **G1** gel can better modulate the fragrance release. So, only this gel was used for the following tests.

### 2.3. Controlled Cleavage of Profragrances **A**–**D**

The lactonization kinetics for the four profragrances **A**–**D**, both in solution and trapped into **G1** was analyzed at 30 and 120 min, exposing the samples prepared in glass vials at the solar light, to have more realistic information on the behavior of these materials in the environment. The analyses were performed in May–June 2022 in Rimini (Italy) selecting only sunny days. The UV index was monitored everyday of exposure for each sample and is reported in Figure S7. Glass containers were used for each sample, as we previously demonstrated that the kinetic is not affected by the material of the container, and glass is commonly used for commercial purposes.

Gels **G1**-**B**, **G1**-**C,** and **G1**-**D** were prepared following the above reported conditions for the preparation of **G1**-**A**, while the corresponding solutions **S**-**A**, **S**-**B**, **S**-**C**, and **S**-**D** were prepared in a 1:1 mixture of H_2_O and ethanol. In all the samples, the concentration of the profragrances was 2.5 mg/mL and the reaction conversion was observed with HPLC-MS analysis as previously reported.

The controlled lactonization was monitored at the same times previously selected for the conversion analysis of **A** with the solar lamp. In this case, the intensity of the UV radiation is variable, as a function of the daytime.

**Figure 8:**
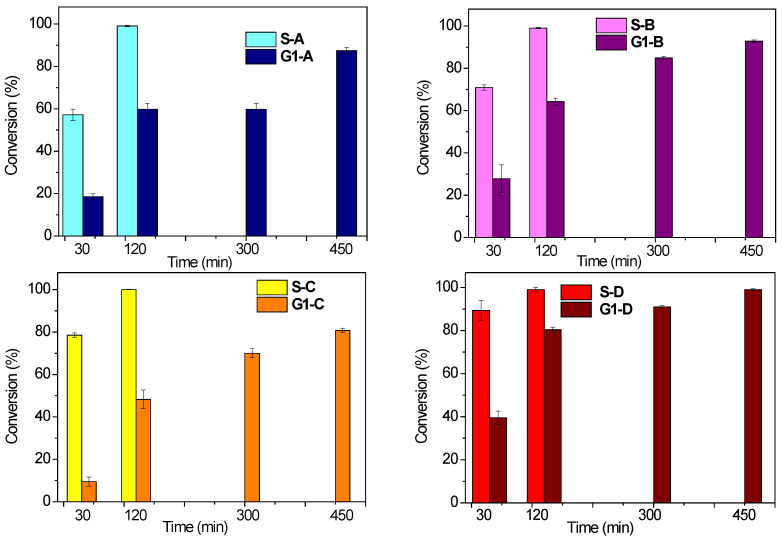
Kinetic of the lactonization of profragrances **A**–**D** in solution (**S**) and in gel (**G1**) using solar light- Top left: profragrance A; top right: profragrance B; bottom left: profragrance C; bottom right: profragrance D. The experiments were repeated in triplicate and results are expressed as mean ± standard deviation.

A general analysis of the results demonstrates that any profragrance trapped in **G1** has reduced lactonization kinetics (Figure 8 and Table S3) compared to the profragrances dissolved in the solvent. Indeed, after 120 min, all the molecules have completely reacted in the solution, while in gel the conversions range between 48.3 and 62.4% for **G1-A**, **G1-B** and **G1-C**. In contrast, the conversion in **G1-D** is 80.4%, which is quite high, even though the shielding effect of the gel is still effective. Thus, the conversion after 300 and 450 min of sun exposal was measured only for gels, and the conversion for **G1-A**, **G1-B**, and **G1-C** was never complete. Only with **G1-D** was the conversion almost 100%. This different behavior may be ascribed to the steric hindrance of the alcohols side chains, which is larger for the molecules containing the aromatic ring (**A**, **B**, and **C**). In addition, **A** and **C** have reduced mobility compared with **B**, and thus their reactivity is further reduced by the gel.

## 3. Conclusions

In this paper, we reported a study on the controlled lactonization of four esters derived from *o*-coumaric acid. The lactonization to coumarin spontaneously occurs after exposure to solar light, releasing coumarin and the corresponding alcohol. All these molecules belong to the family of fragrances. To enhance the persistency of the odor, it is very important to increase the time of fragrance release by slow lactonization of the profragrance. For this aim, we compared the reaction in solution and in gel and we could demonstrate that in gel the lactonization reaction always occurs slower. We also studied which is the more suitable gel for this aim, by comparing the properties of two supramolecular gels obtained with the gelator Boc-L-DOPA(Bn)_2_-OH in a 1:1 ethanol/water mixture in different gelator concentration (0.2% and 1% *w*/*v*). The gel prepared with 1% *w*/*v* gelator concentration is stronger and less transparent than the other gel, and the SEM analysis of the corresponding xerogel reveals that the fibers pack in a very efficient way. We compared the controlled lactonization of one fragrance in both gels and we could demonstrate that these gels better control the process. So, we used the 1% *w*/*v* gel for the other three fragrances. In any case, we obtained a significative reduction of the lactonization reaction in gel compared with the same reaction in solution.

## 4. Materials and Methods

### 4.1. General Remarks for the Synthetic Procedure

All reactions were carried out in dried glassware. The melting points of the compounds were determined in open capillaries and are uncorrected. All compounds were dried in vacuo and all the sample preparations were performed in a nitrogen atmosphere.

High quality infrared spectra (64 scans) were obtained at 2 cm^−1^ resolution with an ATR-IR Agilent (Santa Clara, CA, USA) Cary 630 FTIR spectrometer. NMR spectra were recorded with a Varian (Palo Alto, CA, USA) Inova 400 spectrometer at 400 MHz (^1^H NMR) and at 100 MHz (^13^C NMR). Chemical shifts are reported in δ values relative to the solvent peak. An Agilent (Santa Clara, CA, USA) 1260 Infinity II liquid chromatograph coupled to a Mass Spectrometer MSD/XT equipped with an electrospray ionization source and operating with a single quadrupole mass analyzer was used to check the purity of compounds. The HPLC was equipped with a Phenomenex Gemini C18 −3μ—110 Å column (40 °C) and H_2_O/CH_3_CN with 0.2% formic acid was used as solvent. The MS was used in positive ion mode, *m*/*z* = 50–2000, fragmentor 70 V.

Diisopropyl azodicarboxylate and triphenylphosphine were purchased from ThermoFisher Scientific (Kandel, Germany), trans-*o*-coumaric acid and L-DOPA were purchased from TCI (Tokyo, Japan), and the four alcohols (benzyl alcohol, phenylethyl, cinnamyl alcohol, 9-decen-1yl alcohol) were provided from Farotti s.r.l. (Rimini, Italy). All the solvents were purchased from Sigma-Aldrich (St. Louis, MO, USA).

### 4.2. General Method for the Preparation of Profragrances **A**–**D**

Diisopropyl azodicarboxylate (0.252 mL, 2 mmol) was dissolved in THF (1.9 mL) and added over 15 min period to a solution of alcohol (1 mmol), trans-*o*-coumaric acid (210 mg, 2 mmol), and triphenylphosphine (336 mg, 2 mmol) in THF (4.5 mL) at room temperature (rt). The reaction was stirred for 1.5 h at rt. After this time, the reaction mixture was diluted with ethyl acetate, and then washed with brine, dried over sodium sulfate, filtered, and concentrated at reduced pressure. The residue was purified by silica gel column chromatography to give a white solid.

**A:** Eluant mixture for chromatography: cyclohexane/ethyl acetate 8/2; yield 98.3% (230.9 mg). Mp 85–86 °C; IR-ATR: 3204, 1674, 1623, 1602, 1587 cm^−1^; ^1^H-NMR (400 MHz, CDCl_3_) δ 5.27 (2H, s, COOC*H_2_*), 6.46 (1H, s, O*H*), 6.69 (1H, d, *J* = 16 Hz, PhC*H* = CH), 6.78 (1H, dd, *J* = 1.2, 8 Hz, H_aromatic_), 6.90 (1H, dt, *J* = 1.2, 7.6 Hz, H_aromatic_), 7.22 (1H, dt, *J* = 1.6, 7.2 Hz, H_aromatic_), 7.30–7.46 (6H, m, H_aromatic_), 8.07 (1H, d, *J* = 16 Hz, PhCH = C*H*); ^13^C-NMR (100 MHz, CDCl_3_) δ 168.17, 155.32, 141.13, 136.02, 131.51, 129.32, 128.59, 128.25, 128.24, 121.62, 120.79, 118.10, 116.39, 66.48; HPLC-MS(ESI): 7.8 min; [M+H^+^]: 255, [M+Na^+^]: 277.

**B:** Eluant mixture for chromatography: cyclohexane/ethyl acetate 9/1; yield 89.1% (230.9 mg). Mp 100–102 °C; IR-ATR: 3192, 1675, 1630, 1598 cm^−1^; ^1^H-NMR (400 MHz, CDCl_3_) δ 3.02 (2H, t, *J* = 8 Hz, CH_2_C*H_2_*Ph), 4.44 (2H, t, *J* = 8 Hz, COOC*H_2_*), 6.63 (1H, d, *J* = 16 Hz, PhC*H* = CH), 6.76 (1H, bs, O*H*), 6.83 (1H, d, *J* = 8 Hz, H_aromatic_), 6.90 (1H, t, *J* = 8 Hz, H_aromatic_), 7.20–7.33 (6H, m, H_aromatic_), 7.45 (1H, dd, *J* = 4, 8 Hz, H_aromatic_), 8.04 (1H, d, *J* = 16 Hz, PhCH = C*H*); ^13^C-NMR (100 MHz, CDCl_3_) δ 168.40, 155.42, 140.92, 137.83, 136.68, 131.50, 129.20, 128.95, 128.53, 126.58, 121.64, 120.70, 118.11, 116.42, 65.22, 35.19, 21.97; HPLC-MS(ESI): 8.2 min; [M+H^+^]: 269, [M+Na^+^]: 291.

**C:** Eluant mixture for chromatography: cyclohexane/ethyl acetate 9/1; yield 86.9% (230.9 mg). Mp 133–136 °C; IR-ATR: 3192, 1670, 1622, 1599, 1587 cm^−1^; ^1^H-NMR (400 MHz, CDCl_3_) δ 4.89 (2H, d, *J* = 6.4 Hz, COOC*H_2_*), 6.36 (1H, dt, *J* = 4, 16 Hz, CH_2_C*H* = CHPh), 6.50 (1H, s, O*H*), 6.69 (2H, d, *J* = 16 Hz, PhC*H* = CH), 6.83(1H, d, *J* = 8 Hz, H_aromatic_), 6.91 (1H, t, *J* = 8 Hz, H_aromatic_), 7.19–7.27 (2H, m, H_aromatic_), 7.32 (2H, t, *J* = 4 Hz, H_aromatic_), 7.40 (2H, m, H_aromatic_), 7.46 (1H, d, *J* = 8 Hz, H_aromatic_), 8.07 (1H, d, *J* = 16 Hz, PhCH = C*H*); ^13^C-NMR (100 MHz, CDCl_3_) δ 168.08, 155.33, 141.01, 136.22, 134.27, 131.50, 129.30, 128.59, 128.05, 126.64, 123.25, 121.66, 120.80, 118.15, 116.41, 65.29; HPLC-MS(ESI): 8.7 min, [M+H^+^]: 281, [M+Na^+^]: 303.

**D**: Eluant mixture for chromatography: cyclohexane/ethyl acetate 9/1; yield 89.7% (173.6 mg). M.p. 57–59 °C; IR-ATR: 3192, 1665, 1639, 1615, 1597, 1557 cm^−1^; ^1^H-NMR (CDCl_3_, 400 MHz): δ 1.28–1.40 (10H, m, H_aliphatic_), 1.70 (2H, m, OCH_2_C*H_2_*), 2.02 (2H, m, *H_2_*C-C = C), 4.20 (2H, t, *J* = 8 Hz, COOC*H_2_*), 4.91 (1H, m, CH = CH*H*), 4.98 (1H, m, CH = CH*H*), 5.79 (1H, ddt, *J* = 8, 12, 17.2 Hz, CH_2_C*H =* CH_2_), 6.55 (1H, bs, O*H*), 6.61 (1H, d, *J* = 16 Hz, PhC*H* = CH), 6.83 (1H, dd, *J* = 1.2, 8 Hz, H_aromatic_), 6.90 (1H, dt, *J* = 1.2, 7.6 Hz, H_aromatic_), 7.22 (1H, dt, *J* = 1.6, 8 Hz, H_aromatic_), 7.45 (1H, dd, *J* = 1.6, 7.6 Hz, H_aromatic_), 8.01 (1H, d, *J* = 16, PhCH = C*H*); ^13^C-NMR (100 MHz, CDCl_3_) δ 175.75, 140.39, 139.17, 136.50, 131.33, 129.18, 121.74, 120.72, 118.54, 116.36, 114.13, 64.83, 33.76, 29.33, 29.20, 29.02, 28.86, 28.69, 25.94; HPLC-MS(ESI): 12.03 min; [M+H^+^]: 303, [M+Na^+^]: 325.

### 4.3. Gel Preparation

The gels used for the fragrance release studies were directly prepared in 2 mL HPLC glass vials and the gels used for the rheological analysis were prepared in 7.0 mL Sterilin Cups^®^. All the gels were left to rest for 16 h at room temperature and they were kept in the dark before their use.

For both 1% and 0.2% *w*/*v* concentrations, the gelator was dissolved in the organic solvent (ethanol) by alternating manual shaking and ultrasound sonication until the dissolution of the compound was achieved (few minutes). To trigger the formation of the gel, Milli-Q^®^ H_2_O was added to the vial and immediately gently swirled to allow the mixing of the two solvents. In the case of the gel containing the profragrances for the release studies, a stock solution of the profragrance in ethanol was prepared and added to the vial that already contained the specific amount of gelator. Then, the gelator was dissolved by ultrasound sonication and, right after, Milli-Q^®^ H_2_O was added to form the gel. The ratio EtOH/H_2_O in all the samples was kept to 1:1 and the final concentration of profragrance in each gel was 2.5 mg/mL.

### 4.4. IR Analysis of the Gels Samples

The gels and the solutions used for the infrared spectra were prepared in 2 mL HPLC glass vials. All the gels were left to rest for 16 h at room temperature after their preparation. A small quantity of the gels was withdrawn with a spatula, paying attention not to break the whole bulk. In the case of the ethanol solution, a few drops were enough to record the spectra. The spectra were recorded using the same instrument reported in Section 4.1.

### 4.5. Optical Microscope Analysis

The optical microscope images were recorded using a Nikon (Minato, Japan) ECLIPSE Ti2 Inverted Research Microscope with a 10× or 20× magnifier. A piece of the gel sample prepared in the Sterilin Cups^®^ was cut with a spatula and analyzed while wet.

### 4.6. Rheological Analysis

The rheological analyses were performed using an Anton Paar (Graz, Austria) MCR102 rheometer. A vane and cup measuring system was used, setting a gap of 2.1 mm. The gels were prepared as described and tested directly in the Thermo Fisher Scientific (Waltham, MA, USA) Sterilin Cup^®^ which fits in the rheometer. Oscillatory amplitude sweep experiments (γ: 0.01−100%) were performed at 23 °C using a constant angular frequency of 10 rad/s. The amplitude sweep was repeated three times for each gel and the resulting points of the graphs are provided as mean ± standard deviation.

### 4.7. SEM Analysis

Scanning electron micrographs were recorded on gold coated samples using a Zeiss (Oberkochen, Germany) LEO 1530 operating with a tension of 5 kV.

### 4.8. X-ray Powder Diffraction Analysis

X-ray powder diffraction (XRPD) measurements were performed with a Malvern Panalytical (Amelo, The Netherlands) PanAnalytical X’Pert Pro diffractometer equipped with X’Celerator detector with Cu Kα radiation. The samples were ground before the measurements. The diffraction profiles were analyzed using the software X’Pert High Score Plus.

### 4.9. Solar Lamp Details

The studies of the kinetic of lactonization with the solar lamp were performed using a xenon arc source to simulate the natural sunlight exposure. A 150-W xenon arc lamp (solar simulator, model 68805, Oriel Corporation, Stratford, CT, USA) was used with a dichroic mirror (Oriel, model 81405) to block visible and IR radiation in order to minimize sample heating.

### 4.10. HPLC Analysis

The calibration curves were built with six or seven different dilutions of the stock solution for each profragrance dissolved in acetonitrile. The stock solutions were made with a known profragrance concentration of 1.25 mg/mL, and then the dilutions were made: 1:10, 1:20, 1:50, 1:100, 1:200, 1:500, 1:1000. Each one was analyzed with the HPLC-MS system to obtain the value of each profragrance peak. Then, the concentration of each solution was plotted versus the related peak area (Figures S5 and S6).

For the fragrance release studies with the solar lamp and with the natural sunlight: after the proper interval of time of exposure, each gel was diluted in 1 mL of acetonitrile, and a 1:10 dilution in acetonitrile was made for the HPLC analysis. In the case of the fragrance release studies in solution: after the proper interval of time of exposure, for each solution, a 1:20 dilution was made for the HPLC-MS analysis. In any case (solar lamp and sunlight, gels, and solutions), the experiments were performed in triplicate and the data presented as mean ± standard deviation. The HPLC-MS used and the analyses conditions are the same as reported in Section 4.1.

## Data Availability

The authors confirm that the data supporting the findings of this study are available within the article and its Supplementary Materials.

## Supplementary Materials

The following supporting information can be downloaded at: https://www.mdpi.com/article/10.3390/gels9040350/s1, IR-ATR, ^1^H NMR, ^13^C NMR spectra and HPLC-MS analysis of compounds A-D; Figure S1: Possible interactions of the gelator molecules by H-bonds; Figure S2: Photographs of the trials for the measurement of the MGC of Boc-L-DOPA(Bn)_2_-OH in a 1:1 mixture of H_2_O and EtOH; Figure S3: FT-IR spectra of the gels G02 and G1, compared with the solution S of the gelator Boc-L-DOPA(Bn)_2_-OH; Figure S4: Optical microscope images of wet samples of G02 and G1; Table S1: Amplitude sweep of samples G02 and G1; Figure S5: Calibration curves to calculate the disappearance of A and B by HPLC-MS; Figure S6: Calibration curves to calculate the disappearance of C and D by HPLC-MS; Table S2: Kinetic of the lactonization of profragrance A under selected conditions using the solar lamp; Figure S7: Curve of the UV index recorded during the sunny days of exposure of the samples to sun light; Table S3: Kinetic of the lactonization of profragrances A-D under selected conditions under the sun light.

## References

[1] Berger R.G., Berger R.G. (2007). Flavours and Fragrances: Chemistry, Bioprocessing and Sustainability.

[2] Sell C.S. (2006). The Chemistry of Fragrances: From Perfumer to Consumer.

[3] Surburg H., Panten J. (2016). Common Fragrance and Flavor Materials: Preparation, Properties and Uses.

[4] Armanino N., Charpentier J., Flachsmann F., Goeke A., Liniger M., Kraft P. (2020). What’s Hot, What’s Not: The Trends of the Past 20 Years in the Chemistry of Odorants. Angew. Chem. Int. Ed..

[5] Kraft P., Bajgrowicz J.A., Denis C., Fráter G. (2000). Odds and Trends: Recent Developments in the Chemistry of Odorants. Angew. Chem. Int. Ed..

[6] Valls A., Castillo A., Porcar R., Hietala S., Altava B., García-Verdugo E., Luis S.V. (2020). Urea-Based Low-Molecular-Weight Pseudopeptidic Organogelators for the Encapsulation and Slow Release of (*R*)-Limonene. J. Agric. Food Chem..

[7] Mishra M.K. (2015). Handbook of Encapsulation and Controlled Release.

[8] Ciriminna R., Pagliaro M. (2013). Sol–gel microencapsulation of odorants and flavors: Opening the route to sustainable fragrances and aromas. Chem. Soc. Rev..

[9] Ghosh S., Praveen V.K., Ajayaghosh A. (2016). The Chemistry and Applications of π-Gels. Annu. Rev. Mater. Res..

[10] Giuri D., Jurković L., Fermani S., Kralj D., Falini G., Tomasini C. (2019). Supramolecular Hydrogels with Properties Tunable by Calcium Ions: A Bio-Inspired Chemical System. ACS Appl. Bio Mater..

[11] Giuri D., Barbalinardo M., Zanna N., Paci P., Montalti M., Cavallini M., Valle F., Calvaresi M., Tomasini C. (2019). Tuning Mechanical Properties of Pseudopeptide Supramolecular Hydrogels by Graphene Doping. Molecules.

[12] Draper E.R., Adams D.J. (2017). Low-Molecular-Weight Gels: The State of the Art. Chem.

[13] Terech P., Weiss R.G. (1997). Low Molecular Mass Gelators of Organic Liquids and the Properties of Their Gels. Chem. Rev..

[14] Estroff L.A., Hamilton A.D. (2004). Water Gelation by Small Organic Molecules Water Gelation by Small Organic Molecules. Chem. Rev..

[15] Dastidar P. (2008). Supramolecular gelling agents: Can they be designed?. Chem. Soc. Rev..

[16] Ravarino P., Di Domenico N., Barbalinardo M., Faccio D., Falini G., Giuri D., Tomasini C. (2022). Fluorine Effect in the Gelation Ability of Low Molecular Weight Gelators. Gels.

[17] Sangeetha N.M., Maitra U. (2005). Supramolecular gels: Functions and uses. Chem. Soc. Rev..

[18] Du X., Zhou J., Shi J., Xu B. (2015). Supramolecular Hydrogelators and Hydrogels: From Soft Matter to Molecular Biomaterials. Chem. Rev..

[19] Li Y., Rodrigues J.M., Tomás H. (2012). Injectable and biodegradable hydrogels: Gelation, biodegradation and biomedical applications. Chem. Soc. Rev..

[20] Ghosh G., Barman R., Sarkar J., Ghosh S. (2019). pH-Responsive Biocompatible Supramolecular Peptide Hydrogel. J. Phys. Chem. B.

[21] Yang X., Zhang G., Zhang D. (2012). Stimuli responsive gels based on low molecular weight gelators. J. Mater. Chem..

[22] Nicastro G., Black L.M., Ravarino P., D’agostino S., Faccio D., Tomasini C., Giuri D. (2022). Controlled Hydrolysis of Odorants Schiff Bases in Low-Molecular-Weight Gels. Int. J. Mol. Sci..

[23] Wei M., Pan X., Rong L., Dong A., He Y., Song X., Li J. (2020). Polymer carriers for controlled fragrance release. Mater. Res. Express.

[24] Lv Y., Zhao Y., Liu Y., Zhou Z., Shen Y., Jiang L. (2022). Self-Assembling Oligo(2-oxazoline) Organogelators for the Encapsulation and Slow Release of Bioactive Volatiles. ACS Omega.

[25] Bratovčić A. (2019). Synthesis of Gel Air Freshener and Its Stability. Technol. Acta.

[26] Kaur R., Kukkar D., Bhardwaj S.K., Kim K.-H., Deep A. (2018). Potential use of polymers and their complexes as media for storage and delivery of fragrances. J. Control. Release.

[27] Lee H., Choi C.-H., Abbaspourrad A., Wesner C., Caggioni M., Zhu T., Weitz D.A. (2016). Encapsulation and Enhanced Retention of Fragrance in Polymer Microcapsules. ACS Appl. Mater. Interfaces.

[28] Griesbeck A.G., Hinze O., Görner H., Huchel U., Kropf C., Sundermeier U., Gerke T. (2012). Aromatic aldols and 1,5-diketones as optimized fragrance photocages. Photochem. Photobiol. Sci..

[29] Zhao D., Jiao X., Zhang M., Ye K., Shi X., Lu X., Qiu G., Shea K.J. (2016). Preparation of high encapsulation efficiency fragrance microcapsules and their application in textiles. RSC Adv..

[30] Hofmeister I., Landfester K., Taden A. (2014). pH-Sensitive Nanocapsules with Barrier Properties: Fragrance Encapsulation and Controlled Release. Macromolecules.

[31] Günay K.A., Berthier D.L., Jerri H.A., Benczédi D., Klok H.-A., Herrmann A. (2017). Selective Peptide-Mediated Enhanced Deposition of Polymer Fragrance Delivery Systems on Human Hair. ACS Appl. Mater. Interfaces.

[32] Tekin R., Bac N., Erdogmus H. (2013). Microencapsulation of Fragrance and Natural Volatile Oils for Application in Cosmetics, and Household Cleaning Products. Macromol. Symp..

[33] Herrmann A. (2007). Controlled Release of Volatiles under Mild Reaction Conditions: From Nature to Everyday Products. Angew. Chem. Int. Ed..

[34] Herrmann A. (2017). Profragrance Chemistry as an Interdisciplinary Research Area and Key Technology for Fragrance Delivery. Chimia.

[35] Herrmann A. (2019). Controlled release of volatile compounds using the Norrish type II reaction. Photochemistry.

[36] Lopez-Sanchez J., Alajarin M., Pastor A., Berna J. (2021). Mechanically Interlocked Profragrances for the Controlled Release of Scents. J. Org. Chem..

[37] Liu M., Yan C., Han J., Guo Z., Zhu W., Xiao Z., Wu Y., Huang J. (2021). pH-activated polymeric profragrances for dual-controllable perfume release. AIChE J..

[38] Irawan C., Nur L., Mellisani B., Arinzani (2019). Hanafi Synthesis and characterization of citral-methylanthranilate schiff base, relationship between synthesis time and some physical properties. Rasayan J. Chem..

[39] Irawan C., Islamiyati D., Utami A., Putri I.D., Putri R.P., Wibowo S. (2020). Aurantiol Schiff base as A Raw Material in Fragrance Industry Synthesized by Simple Condensation Method and Its Characterization Using GC-MS. Orient. J. Chem..

[40] Barmatov E., Hughes T. (2021). Degradation of a Schiff-Base corrosion inhibitor by hydrolysis, and its effects on the inhibition efficiency for steel in hydrochloric acid. Mater. Chem. Phys..

[41] Indradas B., Hansen C., Palmer M., Womack G.B. (2014). Autoxidation as a trigger for the slow release of volatile perfumery chemicals. Flavour Fragr. J..

[42] Yang Y., Wahler D., Reymond J.-L. (2003). β-Amino Alcohol Properfumes. Helvetica Chim. Acta.

[43] Derrera S., Flachsmann F., Plessis C. (2007). Melanie Stang Applied Photochemistry—Light Controlled Perfume Release. Chimia.

[44] Nishimura K., Takenaka Y., Kishi M., Tanahashi T., Yoshida H., Okuda C., Mizushina Y. (2009). Synthesis and DNA Polymerase a a and b b Inhibitory Activity of Alkyl P-Coumarates and Related Compounds Chart 1. Chem. Pharm. Bull..

[45] Zanna N., Iaculli D., Tomasini C. (2017). The effect ofl-DOPA hydroxyl groups on the formation of supramolecular hydrogels. Org. Biomol. Chem..

[46] Gaucher A., Dutot L., Barbeau O., Hamchaoui W., Wakselman M., Mazaleyrat J.-P. (2005). Synthesis of terminally protected (S)-β3-H-DOPA by Arndt–Eistert homologation: An approach to crowned β-peptides. Tetrahedron Asymmetry.

[47] Giuri D., Ravarino P., Tomasini C. (2021). l-Dopa in small peptides: An amazing functionality to form supramolecular materials. Org. Biomol. Chem..

[48] Di Filippo M., Giuri D., Marchiori G., Maglio M., Pagani S., Fini M., Tomasini C., Panzavolta S. (2022). Self-assembling of fibers inside an injectable calcium phosphate bone cement: A feasibility study. Mater. Today Chem..

[49] Toronyi A., Giuri D., Martiniakova S., Tomasini C. (2023). Low-Molecular-Weight Gels as Smart Materials for the Enhancement of Antioxidants Activity. Cosmetics.

[50] Pour S.R.S., Oddis S., Barbalinardo M., Ravarino P., Cavallini M., Fiori J., Giuri D., Tomasini C. (2023). Delivery of Active Peptides by Self-Healing, Biocompatible and Supramolecular Hydrogels. Molecules.

[51] Suzuki M., Yumoto M., Shirai H., Hanabusa K. (2008). Supramolecular Gels Formed by Amphiphilic Low-Molecular-Weight Gelators ofNα,Nɛ-Diacyl-L-Lysine Derivatives. Chem. A Eur. J..

[52] Denzer B.R., Kulchar R.J., Huang R.B., Patterson J. (2021). Advanced Methods for the Characterization of Supramolecular Hydrogels. Gels.

[53] Adhikari B., Palui G., Banerjee A. (2009). Self-assembling tripeptide based hydrogels and their use in removal of dyes from waste-water. Soft Matter.

