# Supplementary material for: Controlled Lactonization of o-Coumaric Esters Mediated by Supramolecular Gels
Source: Gels. 2023 Apr 21;9(4):350. doi: 10.3390/gels9040350 (PMC10137337; doi:10.3390/gels9040350)
Supplement: Supplementary file 1 [file gels-09-00350-s001.zip › gels-2354152-supplementary.pdf]

SUPPORTING INFORMATION

Controlled Lactonization of *o*-Coumaric Esters Mediated by Supramolecular Gels

Fabia Cenciarelli, Giuseppe Falini, Demetra Giuri * and Claudia Tomasini *

Dipartimento di Chimica Giacomo Ciamician, Università di Bologna, Via Selmi, 2, 40126 Bologna, Italy

IR-ATR, ^{1}H NMR, ^{13}C NMR spectra and HPLC-MS analysis of compounds **A-D**	Page S2
**Figure S1.** Possible interactions of the gelator molecules by H-bonds.	Page S10
**Figure S2.** Photographs of the trials for the measurement of the MGC of Boc-L-DOPA(Bn)$_2$-OH in a 1:1 mixture of H_2O and EtOH.	Page S11
**Figure S3.** FT-IR spectra of the gels **G02** and **G1**, compared with the solution **S** of the gelator Boc-L-DOPA(Bn)$_2$-OH.	Page S11
**Figure S4.** Optical microscope images of wet samples of **G02** and **G1**	Page S12
**Table S1.** Amplitude sweep of samples **G02** and **G1**	Page S12
**Figure S5.** Calibration curves to calculate the disappearance of **A** and **B** by HPLC-MS.	Page S13
**Figure S6.** Calibration curves to calculate the disappearance of **C** and **D** by HPLC-MS.	Page S14
**Table S2.** Kinetic of the lactonization of profragrance **A** under selected conditions using the solar lamp	Page S15
**Figure S7.** Curve of the UV index recorded during the sunny days of exposure of the samples to sun light	Page S16
**Table S3.** Kinetic of the lactonization of profragrances **A-D** under selected conditions under the sun light	Page S17

IR spectrum of **A:**

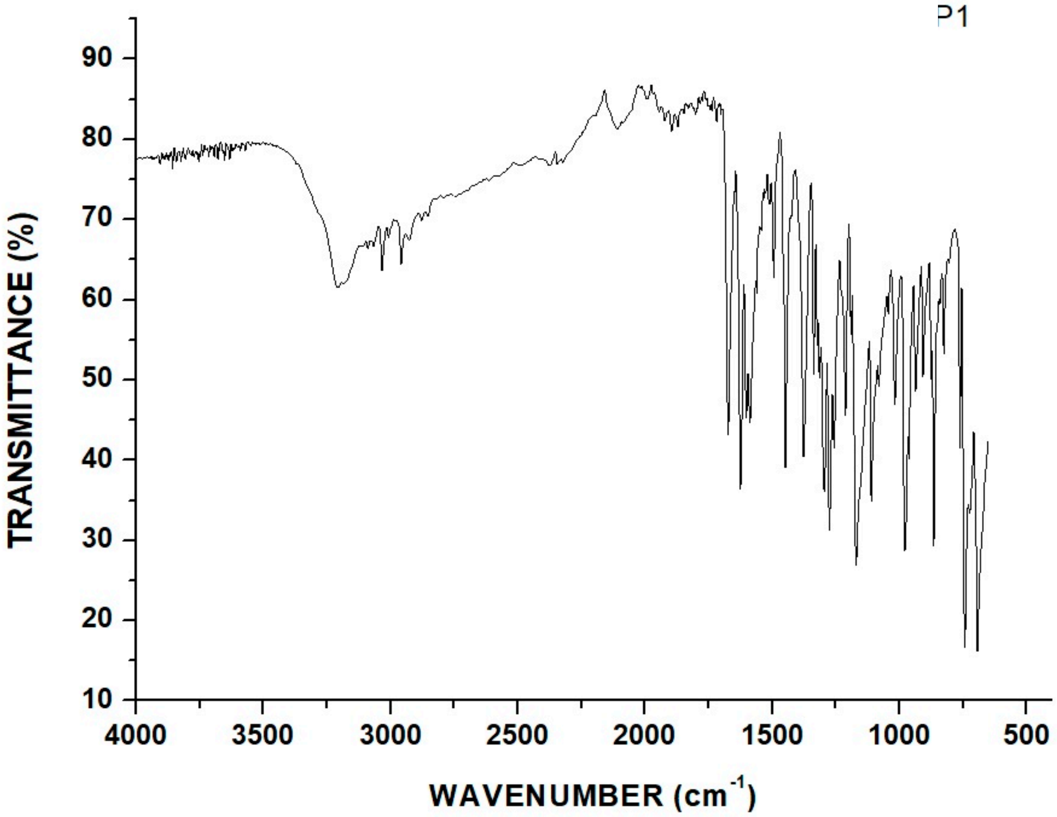

^{1}H-NMR spectrum of **A:**

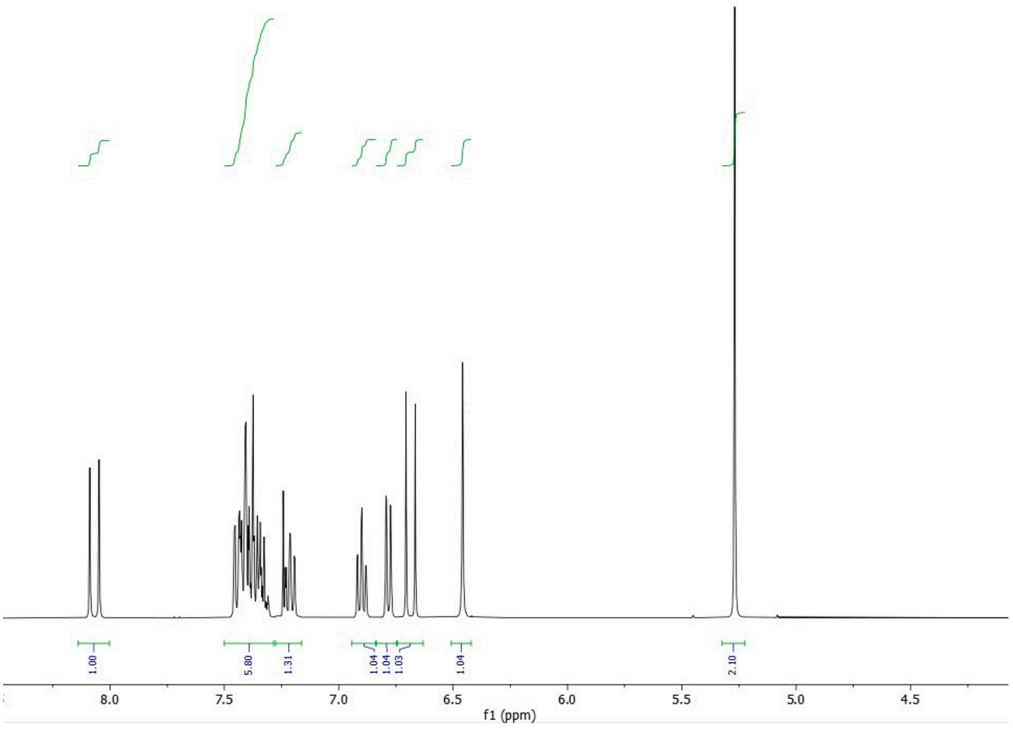

^{13}C-NMR spectrum of **A:**

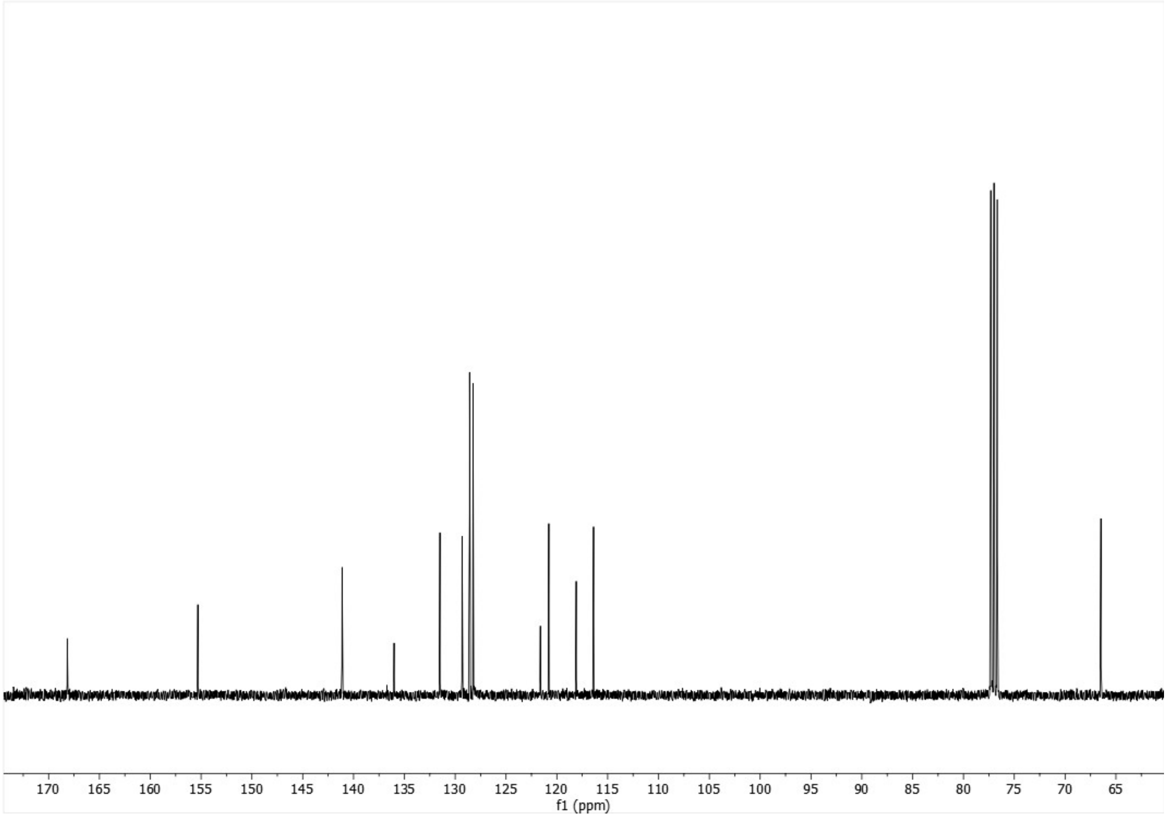

HPLC-MS of **A:**

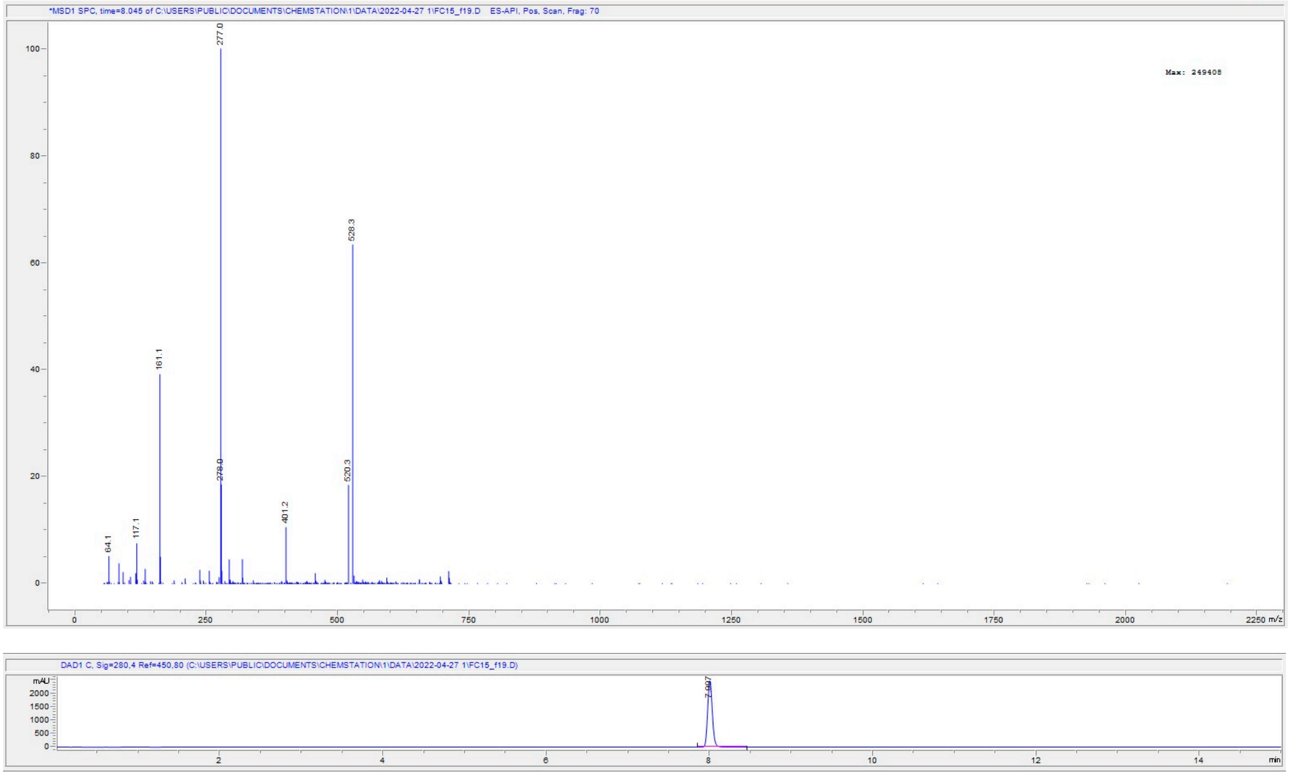

IR spectrum of **B:**

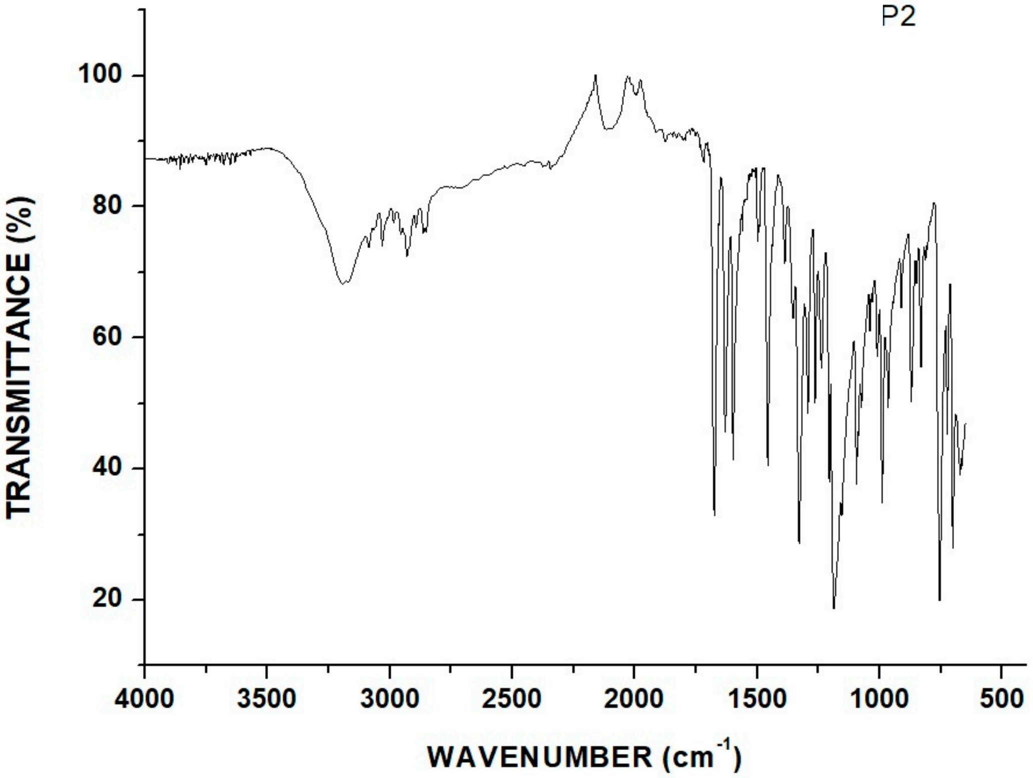

^{1}H-NMR spectrum of **B:**

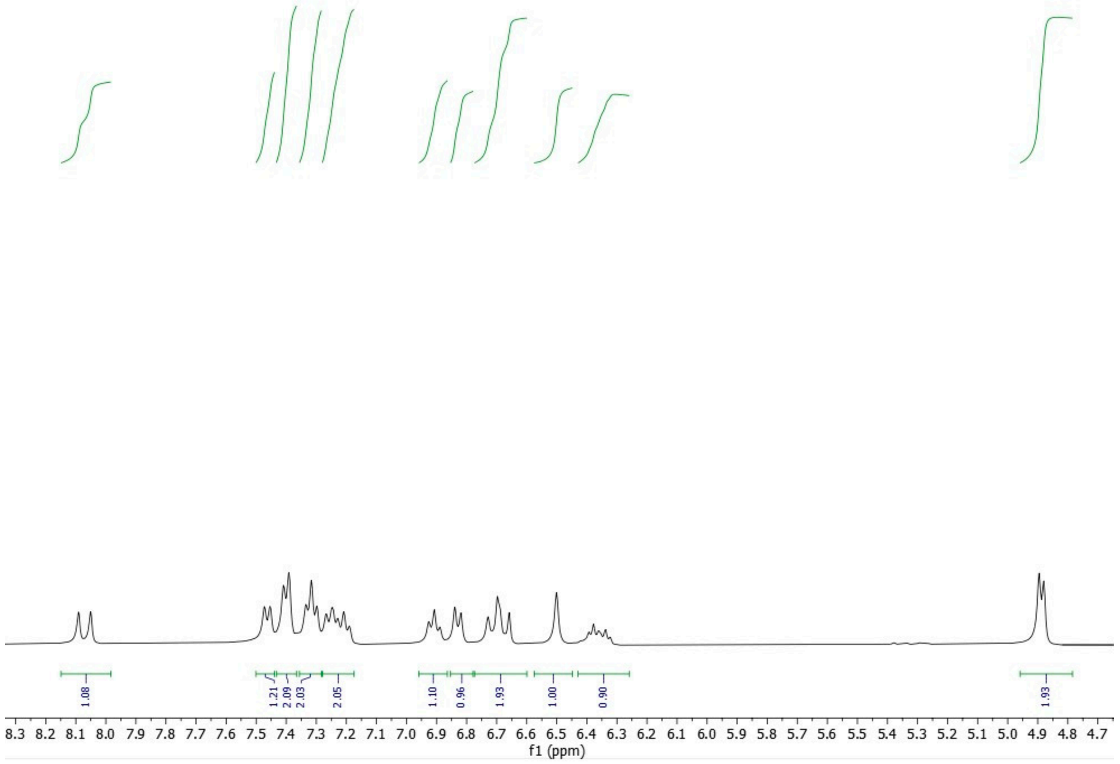

^{13}C-NMR spectrum of **B:**

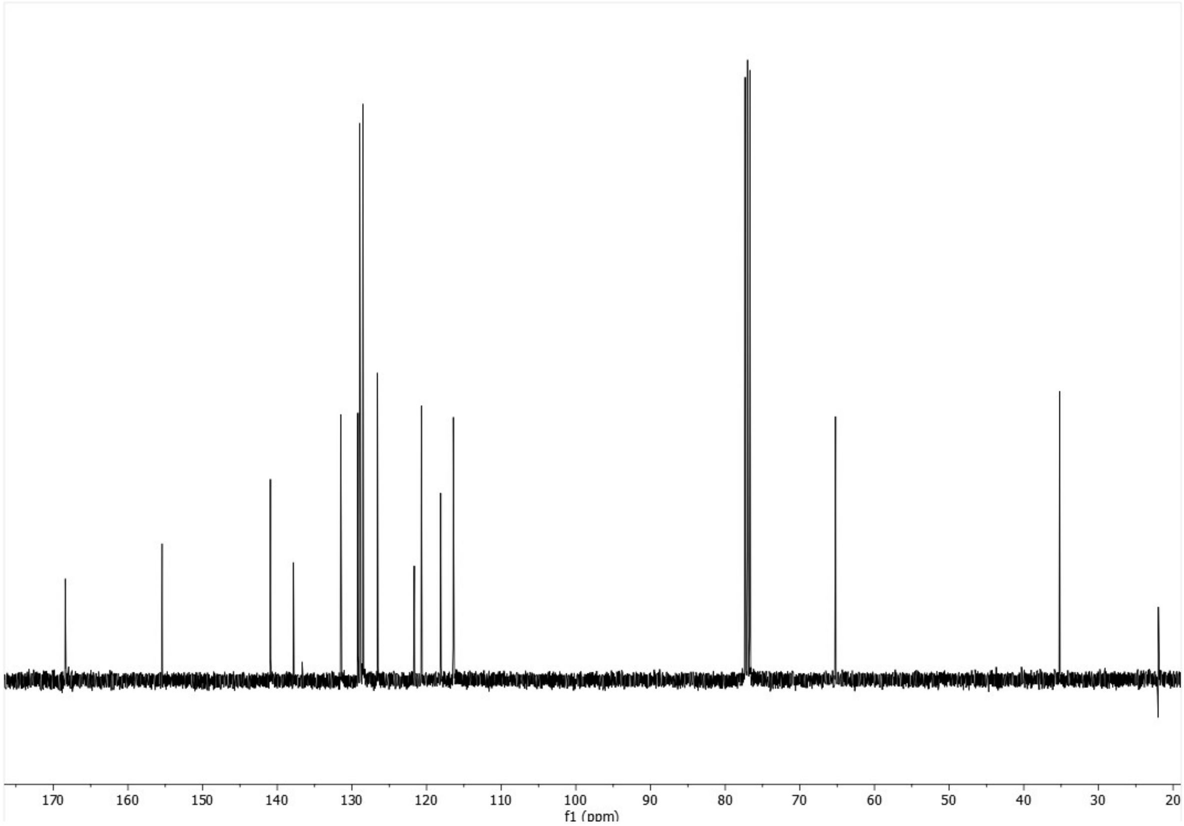

HPLC-MS of **B:**

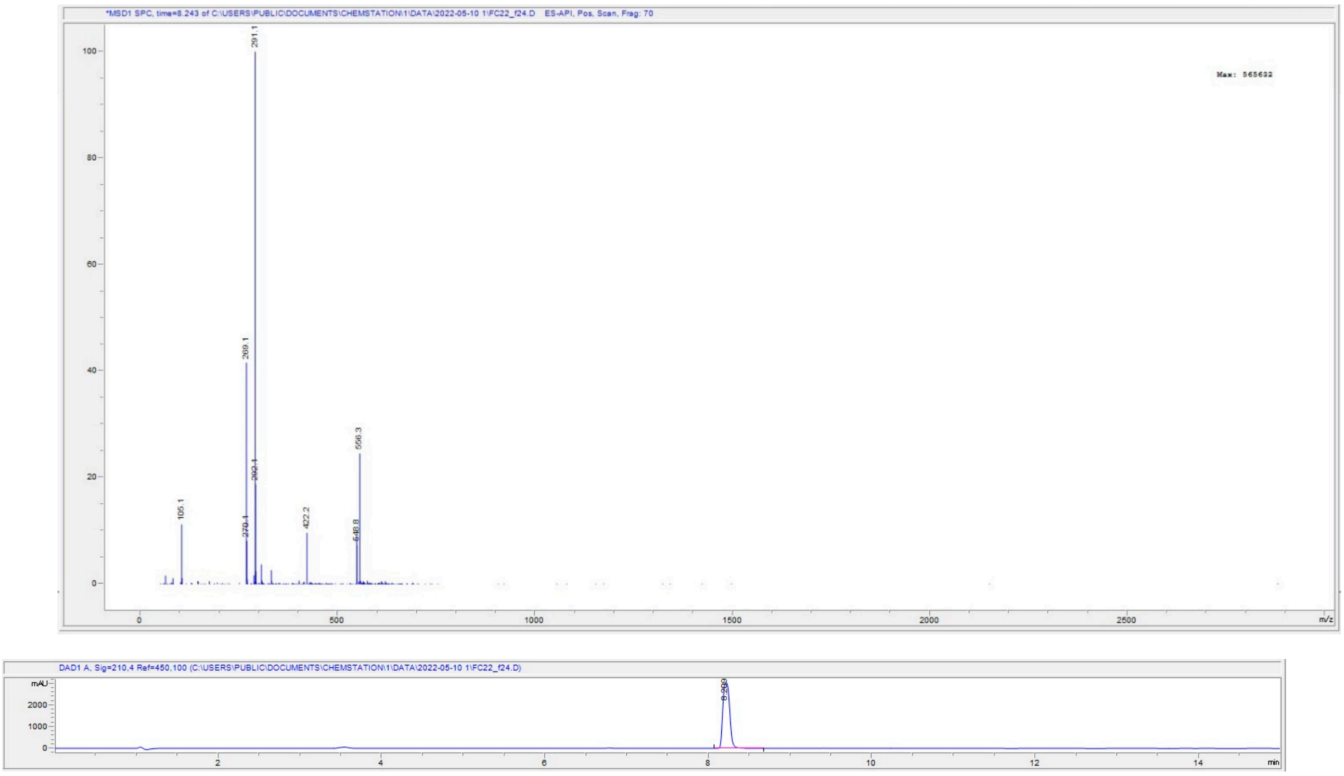

IR spectrum of **C:**

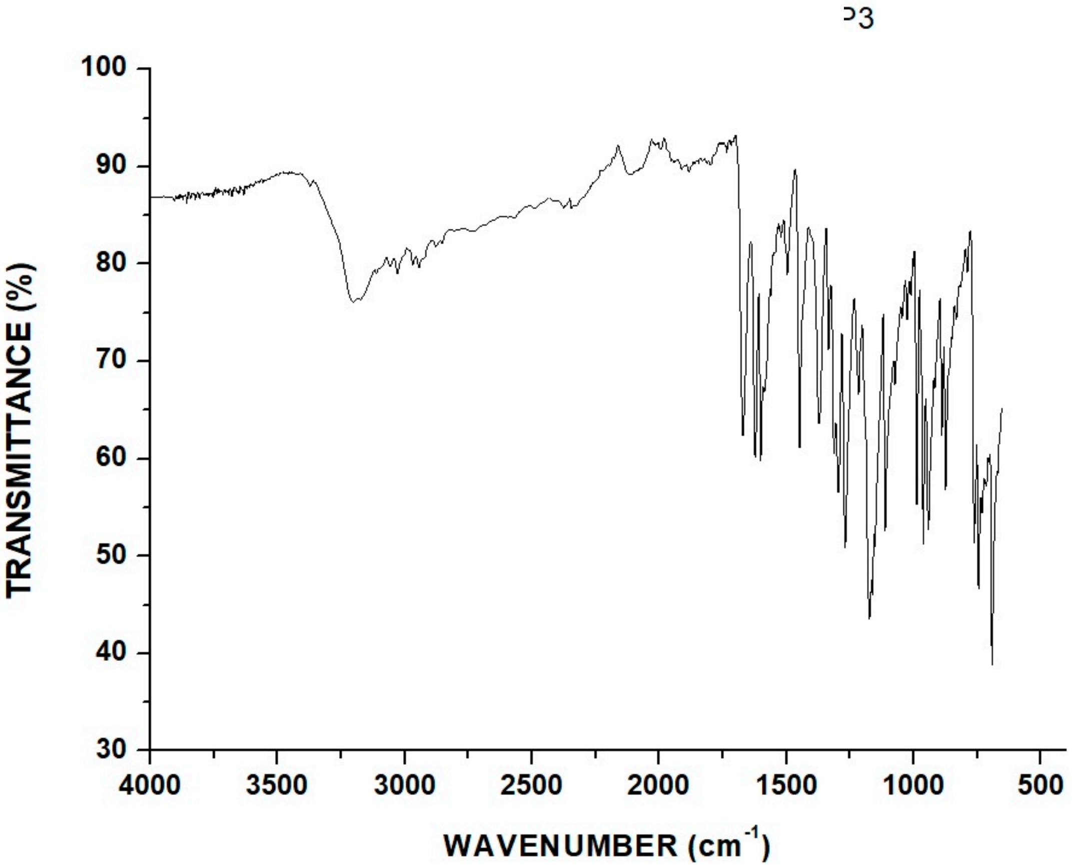

^{1}H-NMR spectrum of **C:**

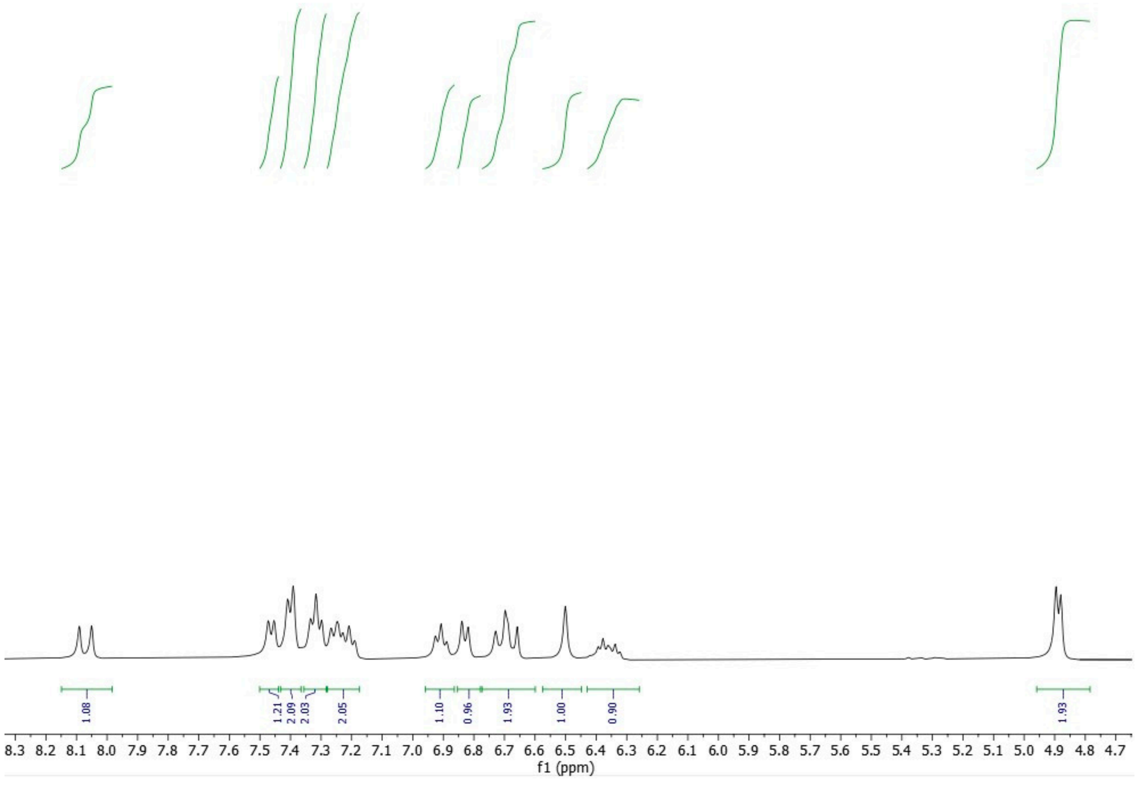

^{13}C-NMR spectrum of **C:**

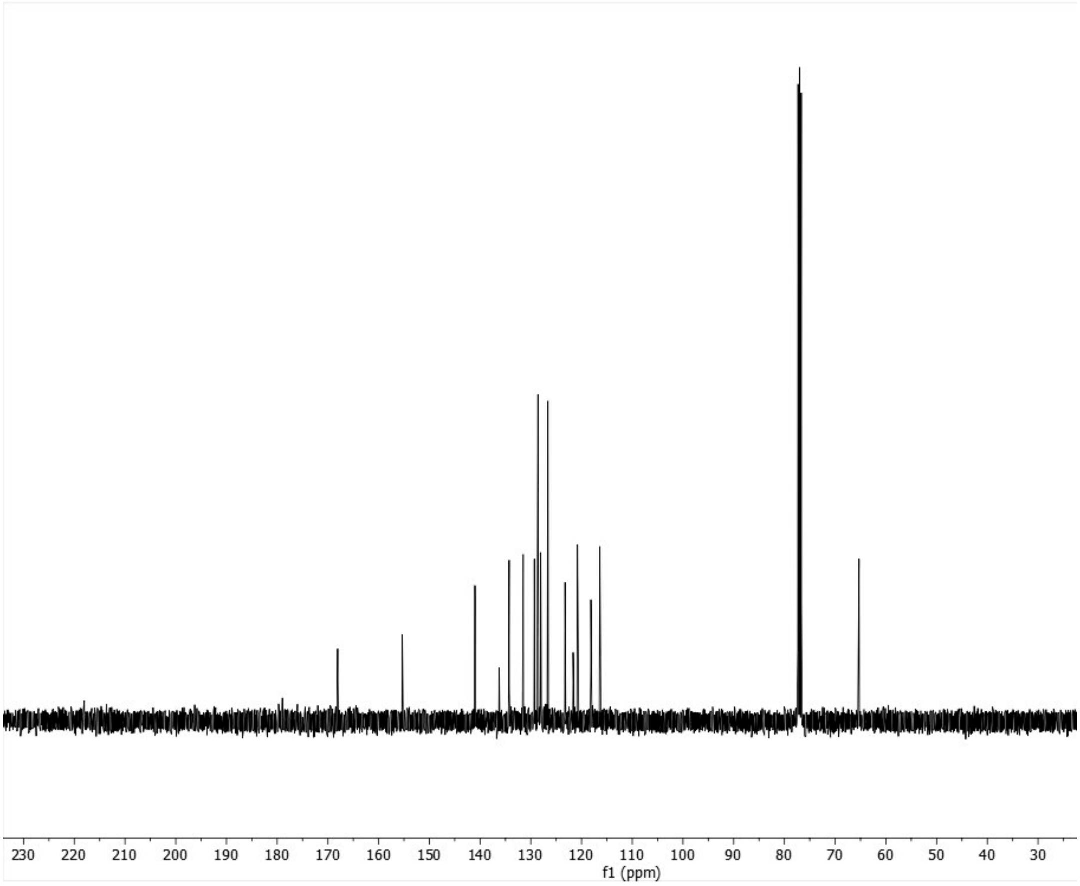

HPLC-MS of **C:**

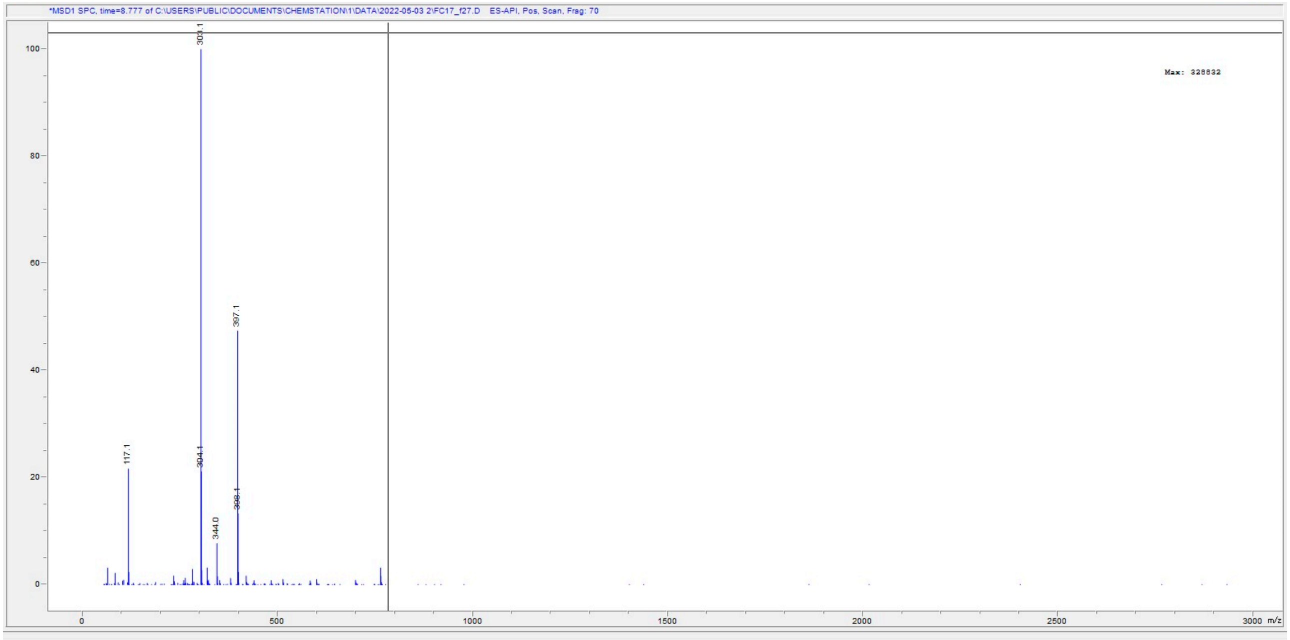

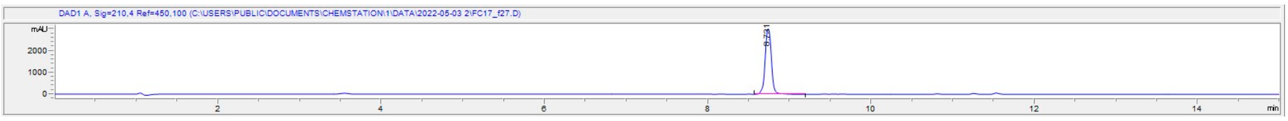

IR spectrum of **D:**

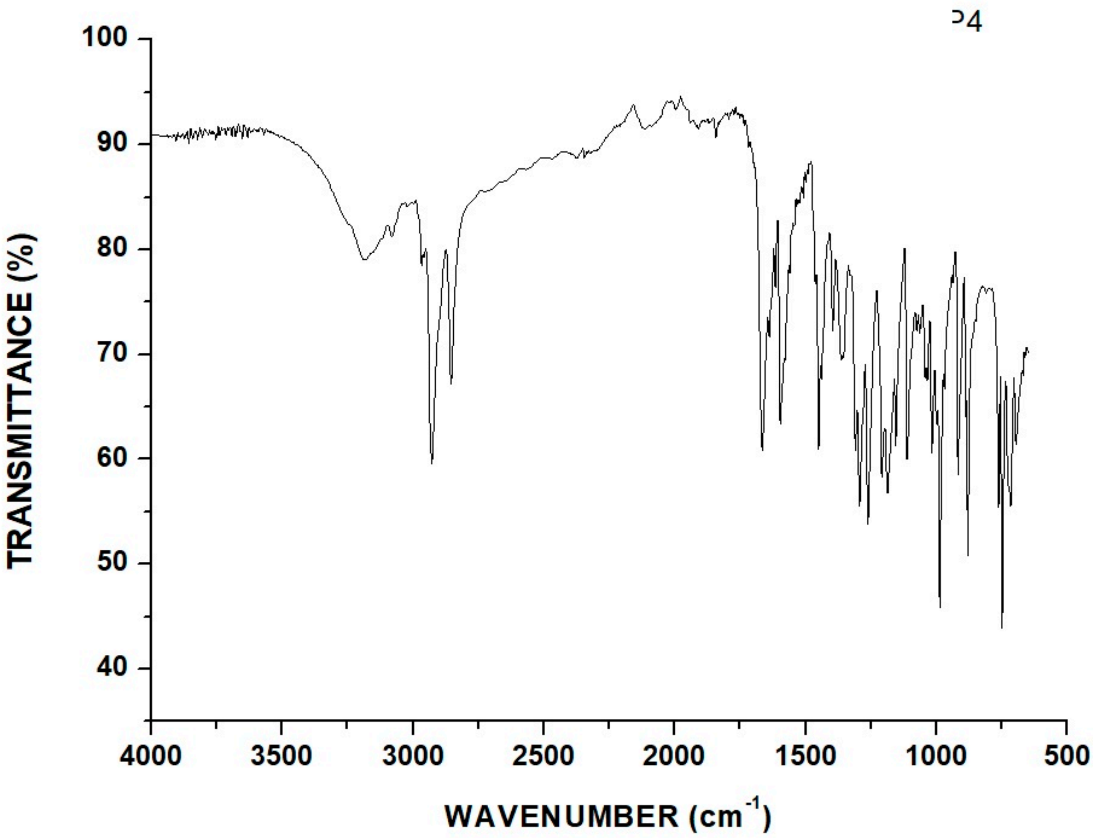

^{1}H-NMR spectrum of **D:**

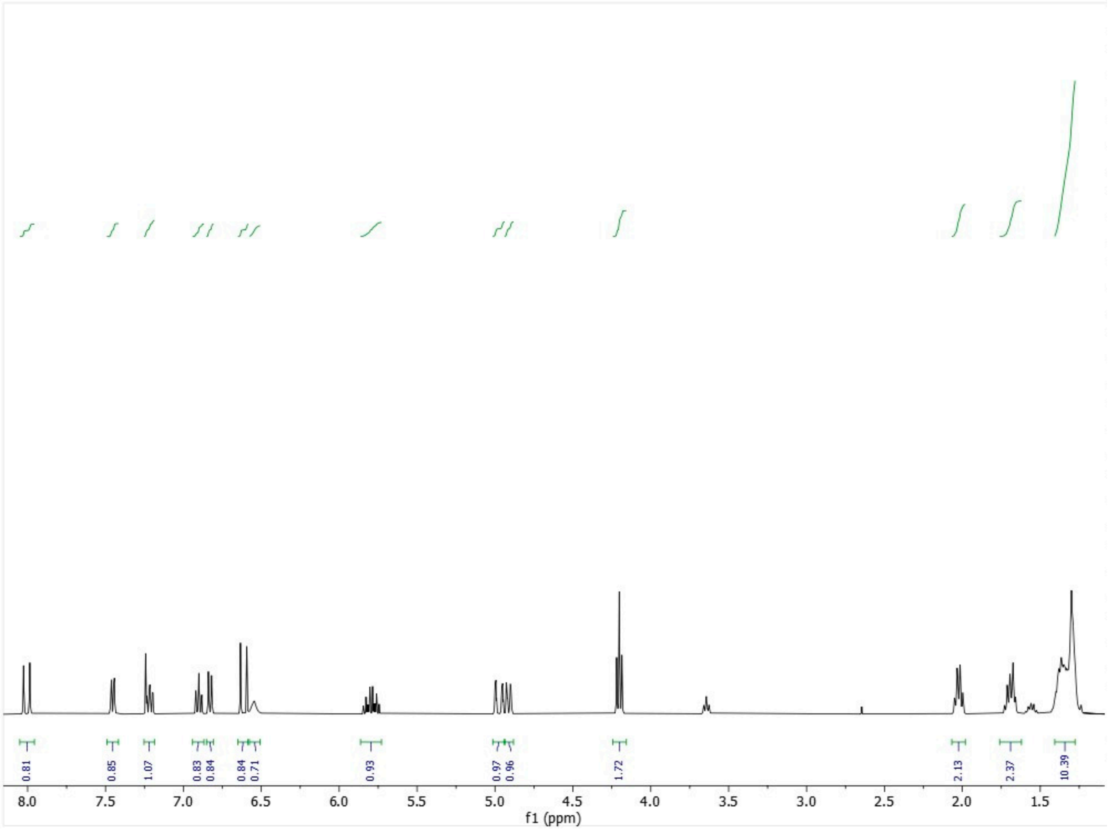

^{13}C-NMR spectrum of **D:**

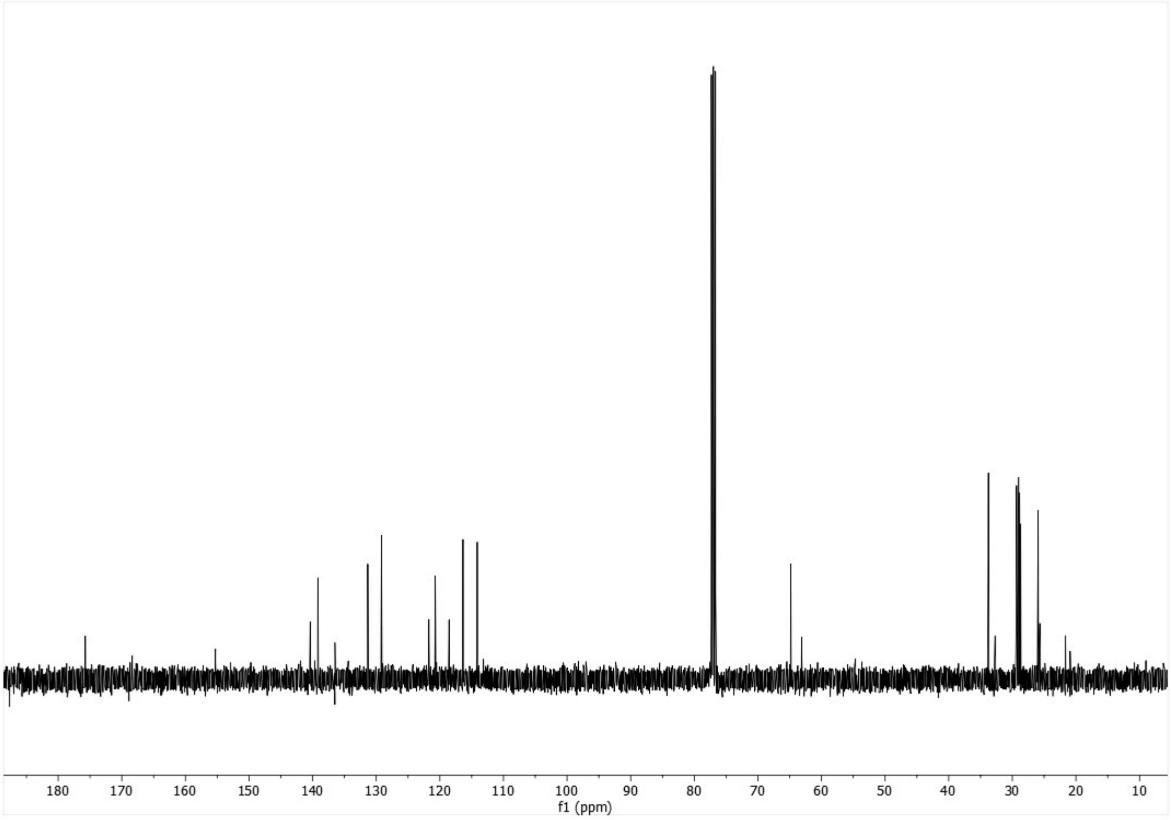

HPLC-MS of **D:**

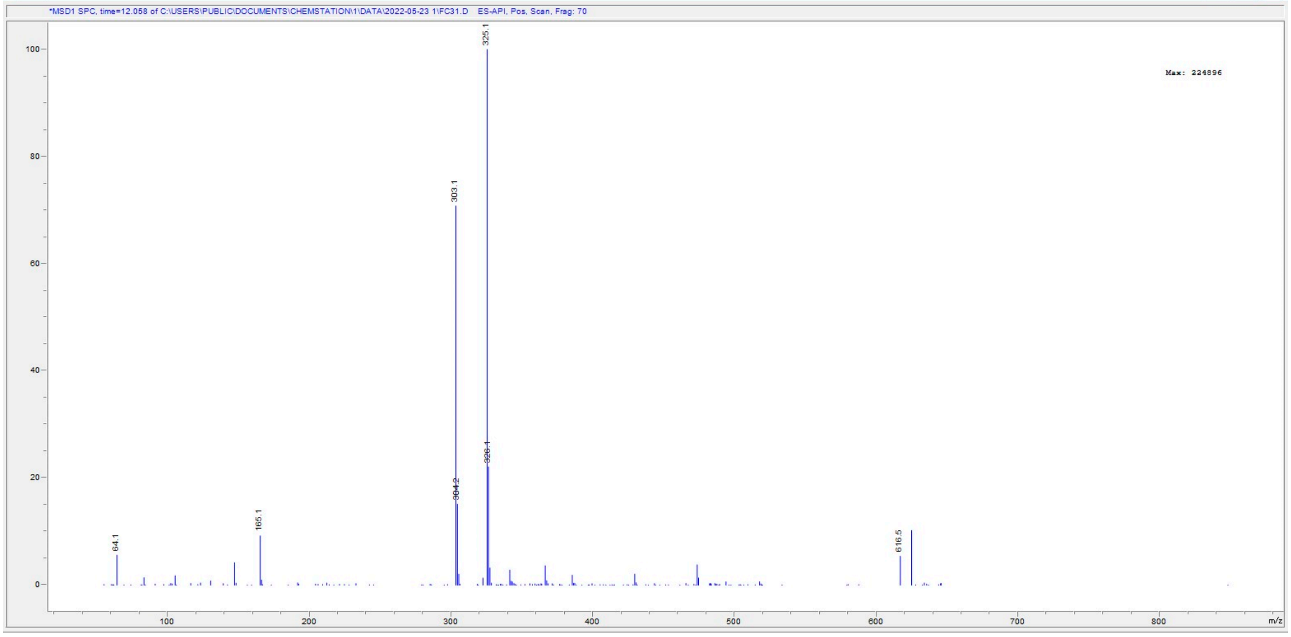

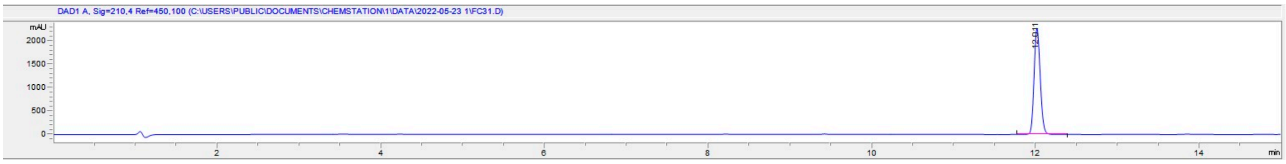

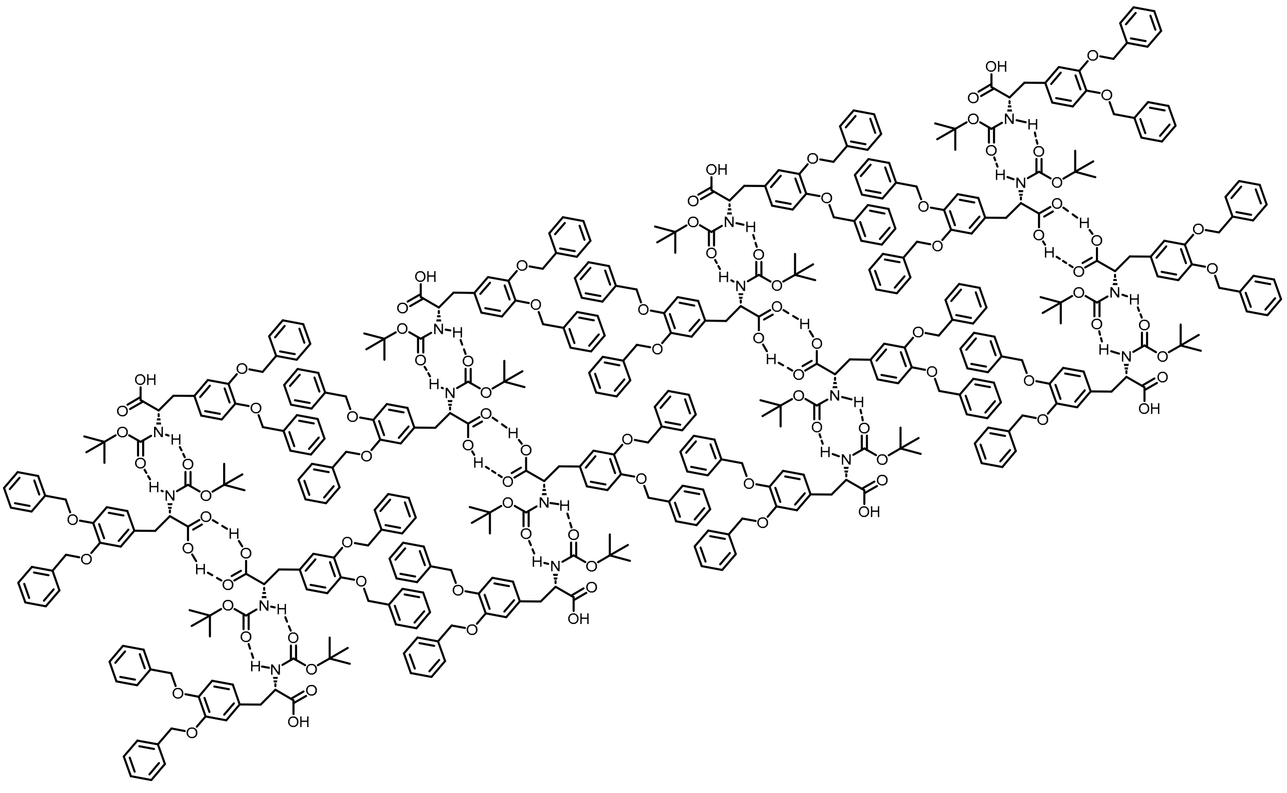

Figure S1. Possible interactions of the gelator molecules by H-bonds.

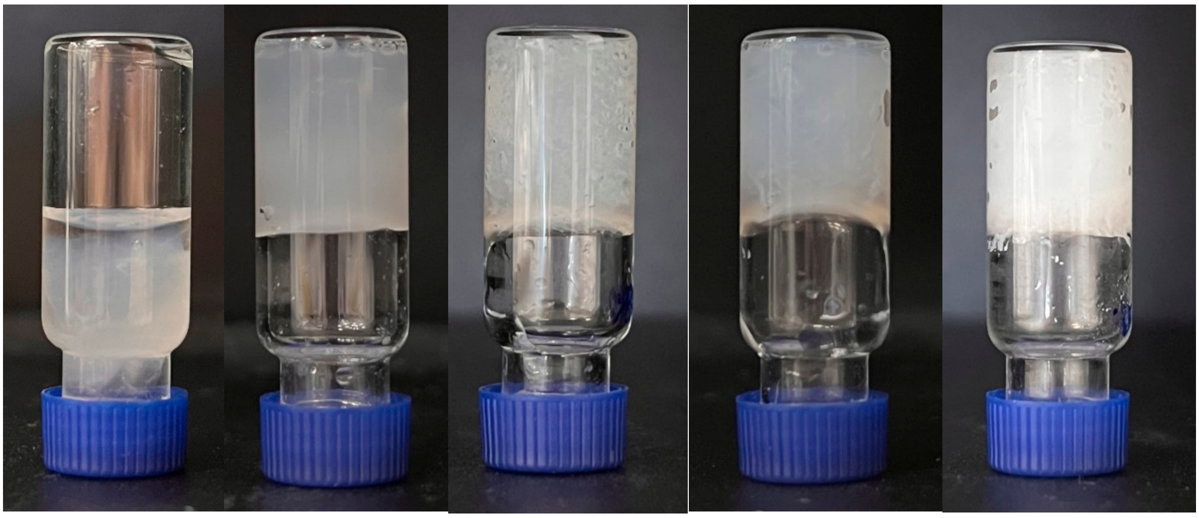

0.1% w/v 0.2% w/v 0.3% w/v 0.5% w/v 1.0% w/v

Figure S2. Photographs of the trials for the measurement of the MGC of Boc-L-DOPA(Bn)$_2$-OH in a 1:1 mixture of H_2O and EtOH. Form left to right: 0.1% w/v, 0.2% w/v, 0.3% w/v, 0.5% w/v, 1.0% w/v concentration. The MGC is 0.2% w/v.

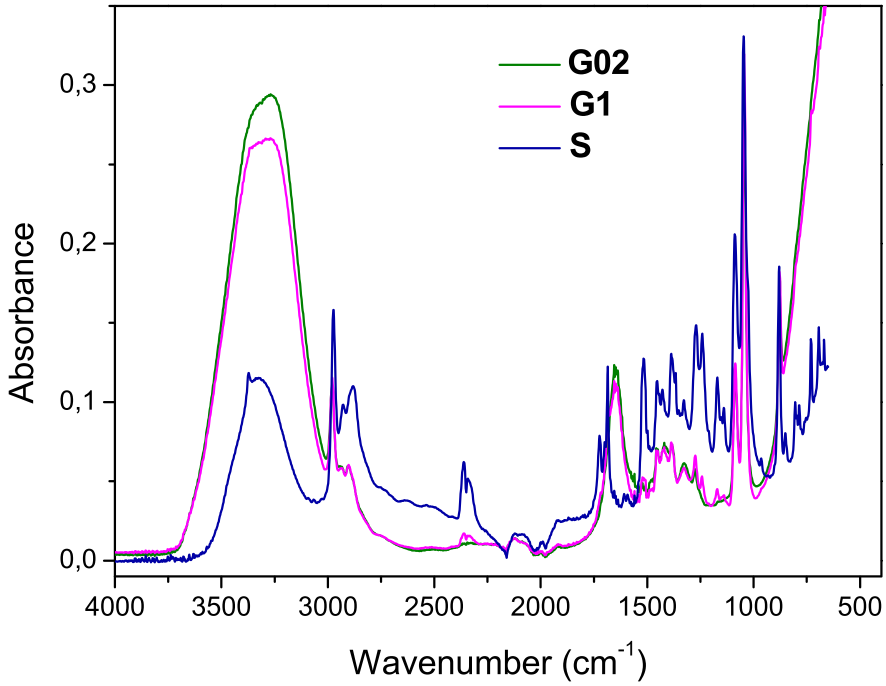

Figure S3. FT-IR spectra of the gels **G02** and **G1**, compared with a solution **S** of the gelator Boc-L-DOPA(Bn)$_2$-OH.

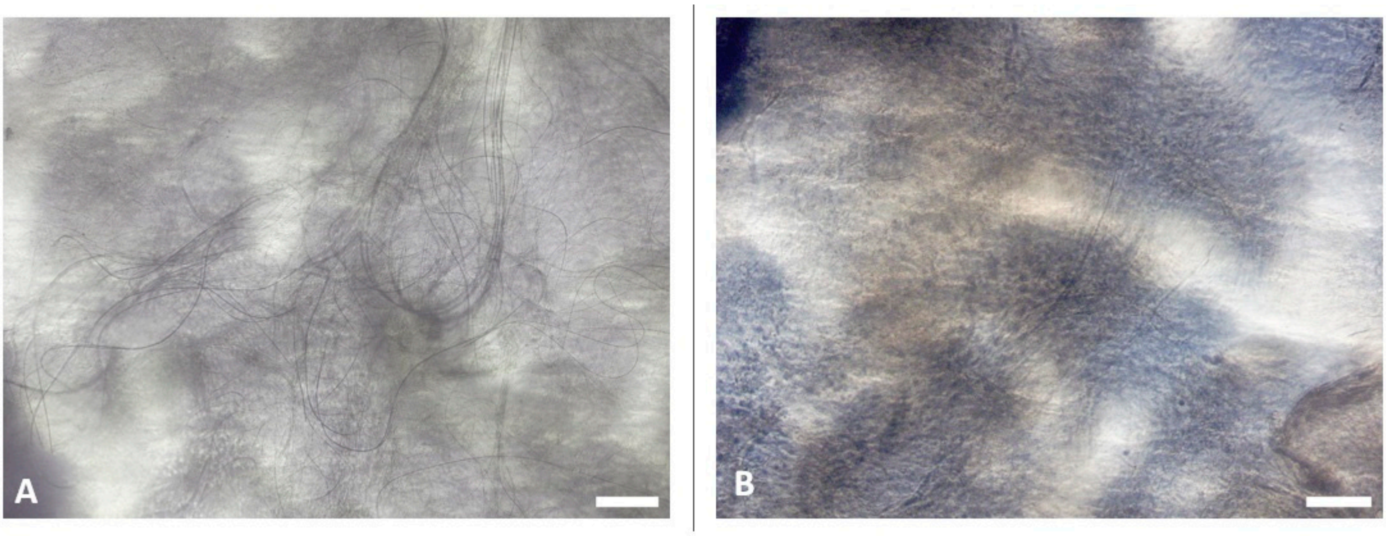

Figure S4. Optical microscope images of Boc-L-DOPA(Bn)$_2$-OH supramolecular gel: (**A**) gelator concentration 0.2% w/v; (**B**) gelator concentration 1% w/v.

Table S1. Storage Modulus (G′), Loss Modulus (G″), Linear Viscoelastic Region (LVER), Crossover Point of hydrogels **G02 and G1**. The experiments were repeated in triplicate and results are expressed as mean ± standard deviation.

Gel	G′ (kPa)	G″ (kPa)	LVER (γ %)	Crossover point (γ %)
**G02**	1.59 ± 0.44	0.38 ± 0.09	0.15	N.D.
**G1**	37.9 ± 2.86	10.2 ± 0.55	0.16	32.1

G′ and G′′ are taken at γ = 0.046 % as at that strain none of the gel has inflections in the trend of their moduli; N.D: non detected.

Calibration curve of **A:**

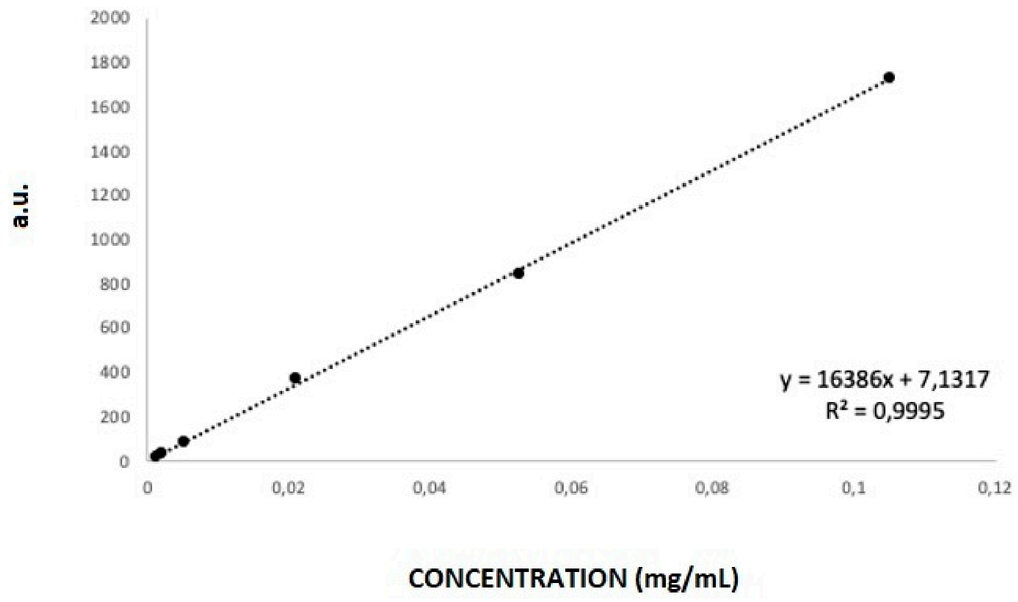

Calibration curve of **B**:

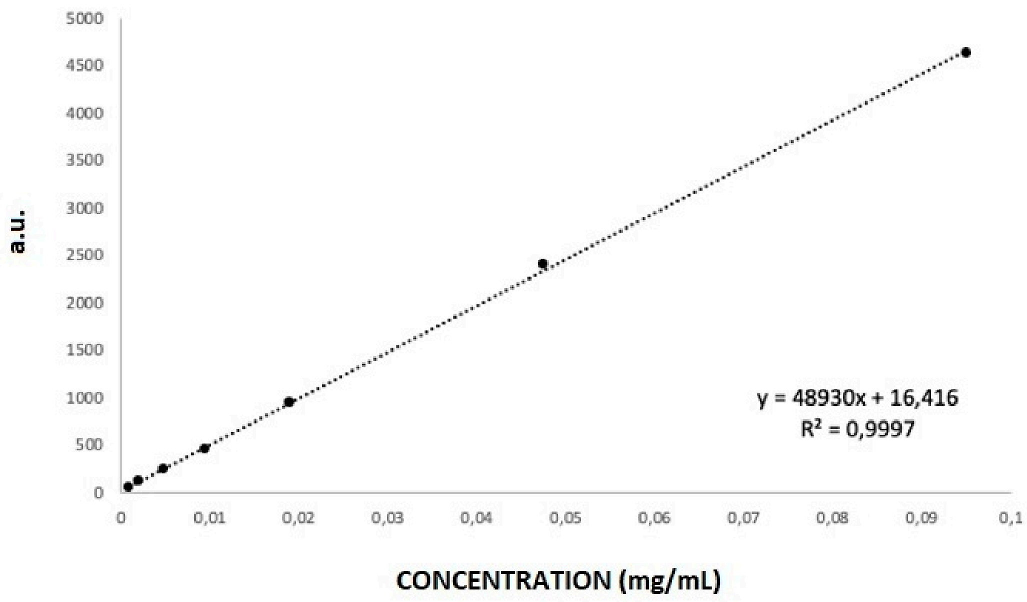

Figure S5. Calibration curves to calculate the disappearance of **A** and **B** by HPLC-MS.

Calibration curve of **C**:

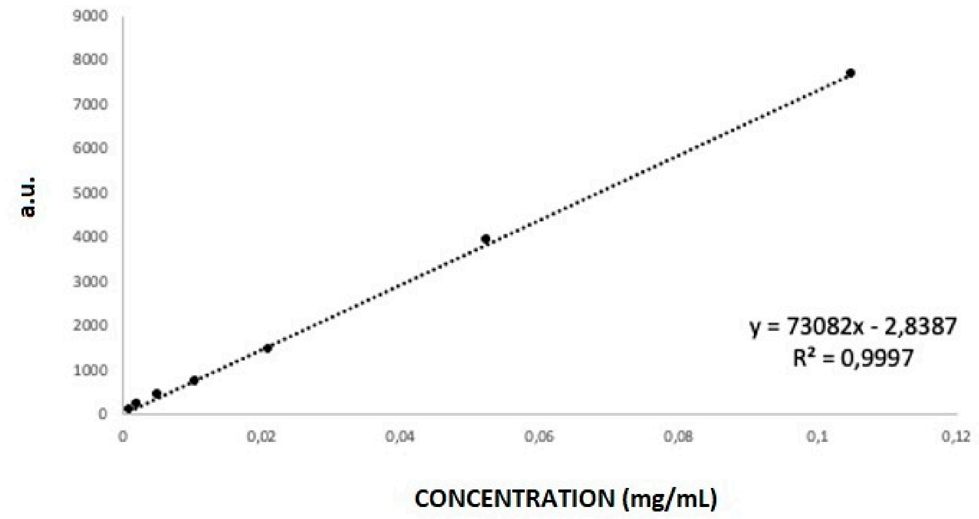

Calibration curve of **D:**

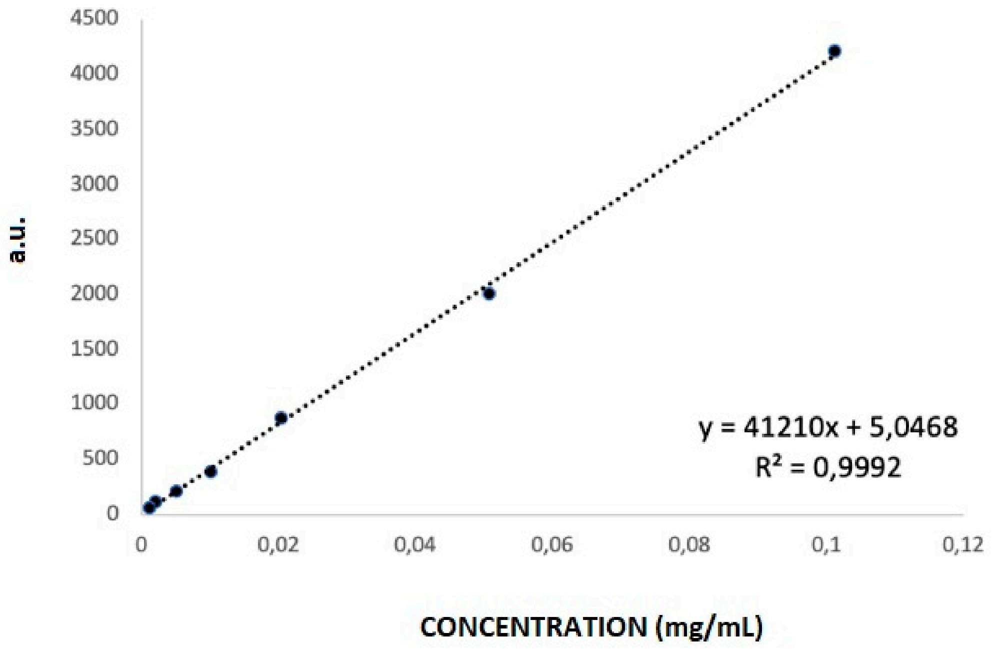

Figure S6. Calibration curves to calculate the disappearance of **C**and **D** by HPLC-MS.

Table S2. Kinetic of the lactonization of profragrance **A** under selected conditions using a solar lamp. The experiments were repeated in triplicate and results are expressed as mean ± standard deviation.

Entry	Sample	Cell material	Time (min)	Conversion (%)
1	**S-A**	quartz	30	67.4 ± 0.958
			120	97.6 ± 0.638
		glass	30	66.6 ± 3.20
			120	96.0 ± 0.145
2	**G02-A**	quartz	30	67.3 ±1.15
			120	81.8 ± 1.37
		glass	30	67.9 ± 0.454
			120	83.3 ± 0.915
3	**G1-A**	quartz	30	56.8 ± 1.23
			120	85.1 ± 1.41
		glass	30	56.3 ± 0.396
			120	80.8 ± 2.24

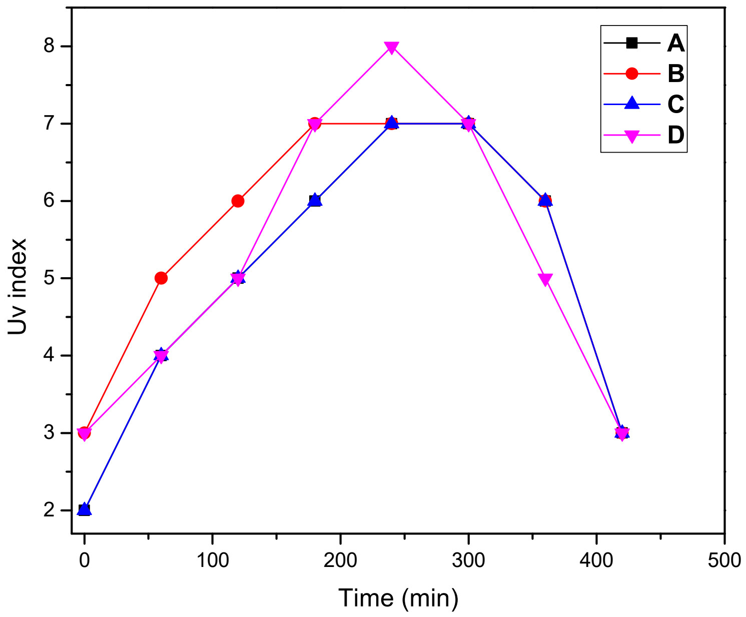

Figure S7. Curve of the UV index recorded during sunny days in June 2022 in Rimini (Italy). After exposure of the samples to sun light over the day (to have the same conditions of UV index for each sample), the analysis of the profragrance concentration were performed in triplicate, both in solutions and in gels.

Table S3. Kinetic of the lactonization of profragrances **A-D** under exposure to solar light at different times in solutions (**S**) and in gels (**G**). The experiments were repeated in triplicate and results are expressed as mean ± standard deviation.

Entry	Sample	Time (min)	Conversion (%)
1	**S-A**	30	86.7 ± 0.716
		120	100 ± 0.00
2	**G1-A**	30	18.5 ± 1.33
		120	59.8 ± 2.79
		300	87.4 ± 1.52
		450	95.3 ± 0.944
3	**S-B**	30	70.9 ± 1.26
		120	100 ± 0.00
4	**G1-B**	30	27.8 ± 6.50
		120	46.3 ± 2.45
		300	77.4 ± 1.02
		450	89.3 ± 0.879
5	**S-C**	30	78.5 ± 1.07
		120	100 ± 0.00
6	**G1-C**	30	9.46 ± 2.17
		120	21.0 ± 6.73
		300	54.2 ± 3.24
		450	70.6 ± 1.65
7	**S-D**	30	89.4 ± 4.52
		120	100 ± 0.00
8	**G1-D**	30	39.5 ± 3.08
		120	72.5 ± 1.68
		300	87.4 ± 0.685
		450	100 ± 0.00
